# A straightforward conversion of 1,4-quinones into polycyclic pyrazoles via [3 + 2]-cycloaddition with fluorinated nitrile imines

**DOI:** 10.3762/bjoc.17.108

**Published:** 2021-06-28

**Authors:** Greta Utecht-Jarzyńska, Karolina Nagła, Grzegorz Mlostoń, Heinz Heimgartner, Marcin Palusiak, Marcin Jasiński

**Affiliations:** 1 Department of Organic and Applied Chemistry, Faculty of Chemistry, University of Lodz, Tamka 12, 91403 Łódź, Poland; 2 Department of Chemistry, University of Zurich, Winterthurerstrasse 190, CH-8057 Zurich, Switzerland; 3 Department of Physical Chemistry, Faculty of Chemistry, University of Lodz, Pomorska 163/165, 90236 Łódź, Poland

**Keywords:** [3 + 2]-cycloadditions, fluorinated compounds, fused pyrazoles, N-heterocycles, nitrile imines, 1,4-quinones

## Abstract

In-situ-generated *N*-aryl nitrile imines derived from trifluoroacetonitrile efficiently react with polycyclic 1,4-quinones, yielding fused pyrazole derivatives as the exclusive products. The reactions proceed via the initially formed [3 + 2]-cycloadducts, which undergo spontaneous aerial oxidation to give aromatized heterocyclic products. Only for 2,3,5,6-tetramethyl-1,4-benzoquinone, the expected [3 + 2]-cycloadduct exhibited fair stability and could be isolated in moderate yield (53%). The presented method offers a straightforward access to hitherto little known trifluoromethylated polycyclic pyrazoles. All products were isolated as pale colored solids with medium-intensity absorption maxima in the range of 310–340 nm for naphthoquinone-derived products and low-intensity bands in the visible region (≈400 nm) for the anthraquinone series.

## Introduction

**Figure 1:**
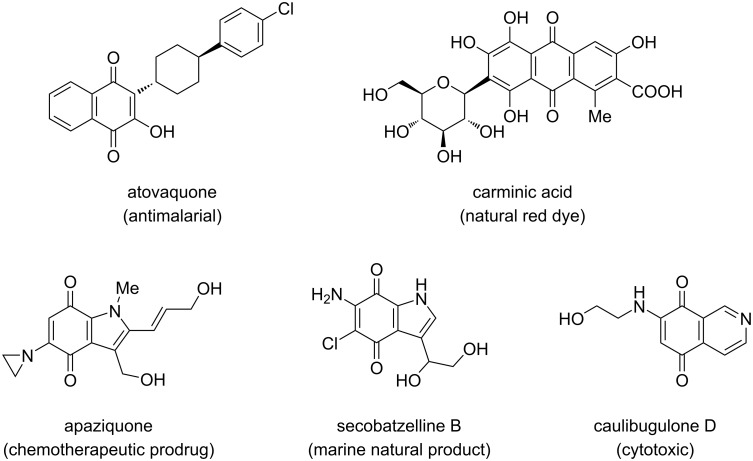
Structures of exemplary benzo- and heteroaromatic fused 1,4-quinone drugs and natural products.

The 1,4-quinone scaffold belongs to the most important structural motifs present in naturally occurring compounds as well as synthetic drugs and other functionalized organic molecules of great practical importance, e.g., in materials chemistry (Figure 1) [1–4].

Selective functionalization of 1,4-quinones is a challenging task in current organic synthesis, and diverse transformations are known to create new C–C bonds and/or to extend the (poly)cyclic system. In this context, cycloadditions are of special importance, and Diels–Alder reactions have successfully been explored in conversions aimed at the construction of a new, six-membered ring [5]. However, [3 + 2]-cycloadditions leading to five-membered heterocycles are less often employed in spite of the high dipolarophilicity of the α,β-unsaturated diketone system [6–9]. Notably, in the already reported reactions of propargylic 1,3-dipoles, such as nitrile oxides or nitrile ylides, with 1,4-quinones, competitive reaction courses involving either ethylenic C=C or carbonyl C=O bonds were observed. For example, the more polar arylnitrile oxides and 1,4-benzoquinones reacted via addition to the C=C bond to give fused isoxazole derivatives [10–12] as well as with the C=O bond yielding spirocyclic 1,4,2-dioxazole derivatives [13–14]. Furthermore, for photochemically generated benzonitrile isopropanide, competitive C=O and C=C additions with 1,4-quinones were observed [15]. On the other hand, the slightly less polar benzonitrile benzylide underwent [3 + 2]-cycloaddition to the C=C bond exclusively [16]. Noteworthy, [3 + 2]-cycloadditions of nitrile imines to the C=O group of 1,4-quinones have not yet been reported.

**Scheme 1:**
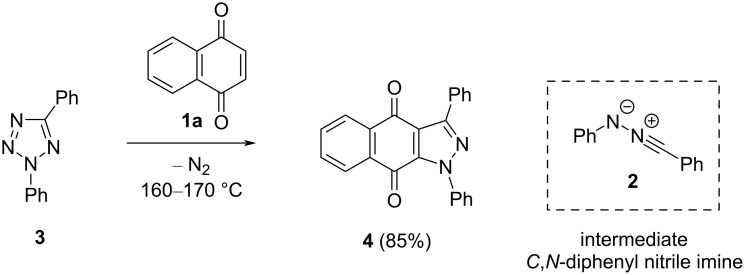
First [3 + 2]-cycloaddition of 1,4-naphthoquinone (**1a**) with intermediate nitrile imine **2**, generated from tetrazole **3**, reported by Huisgen et al. [17].

In a historical work by Rolf Huisgen et al., the first [3 + 2]-cycloadditions of some 1,4-quinones, e.g., 1,4-naphthoquinone (**1a**), with *C*,*N*-diphenyl nitrile imine (**2**) were reported in the 1960s [17]. The latter 1,3-dipole was generated thermally from the respective tetrazole derivative **3**, and the observed [3 + 2]-cycloadditions occurred chemoselectively to provide fused pyrazoles of the type **4** as exclusive products (Scheme 1).

This type of cycloaddition attracted considerable attention of other groups [18–19] and recently has been applied for the preparation of some π-extended pyrazole derivatives, which exhibited promising biological activity [20].

**Scheme 2:**
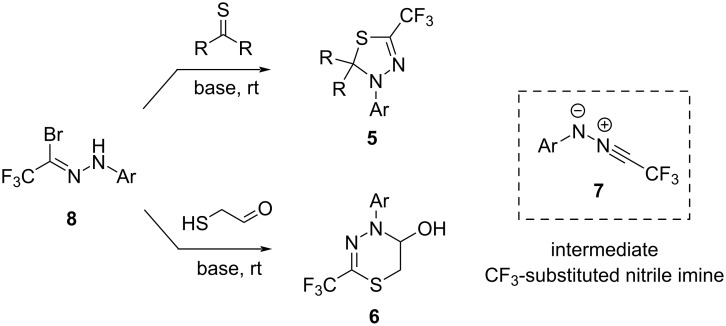
Selected applications of trifluoroacetonitrile imines **7** in the synthesis of S-containing 5- and 6-membered heterocycles.

In a series of our recent publications, efficient syntheses of fluoromethylated five- and six-membered N,S-heterocycles, such as **5** and **6**, available via [3 + 2]-cycloadditions [21–25] or [3 + 3]-annulations [26] of trifluoroacetonitrile imines **7**, respectively, were reported (Scheme 2).

Noteworthy, the latter 1,3-dipoles are easily generated under mild conditions via base-mediated dehydrobromination of the respective hydrazonoyl bromides **8** and smoothly undergo [3 + 2]-cycloadditions with both electron-rich C=C dipolarophiles [27–29] and arynes [30], yielding the corresponding pyrazole derivatives. Unexpectedly, they reacted also with electron-deficient polyfluorinated thioamides to give the desired 1,3,4-thiadiazoles as the products of [3 + 2]-cycloaddition to the C=S bond [25].

Taking into account that fluorinated heterocycles [31–34], including pyrazoles [31,35–36], are of great significance for various medicinal and agricultural applications, the development of new methods for the construction of fluorine-containing organic molecules combined with the 1,4-quinone moiety can be considered as a challenging problem of current organic synthesis. Thus, the main goal of the present study was to check the course of [3 + 2]-cycloaddition reactions of electron-deficient CF_3_-substituted nitrile imines **7** with 1,4-naphthoquinone (**1a**) and 1,4-anthraquinone (**1b**), which were selected as model dipolarophiles. In addition, an important issue of the work was the examination of the chemoselectivity governing the formation of five-membered rings via competitive cycloaddition of the in-situ-generated 1,3-dipoles either onto the C=C or C=O bond. The present work should also be considered as an extension of our earlier studies focused on the exploration of 1,4-quinones in the [3 + 2]-cycloaddition and hetero-Diels–Alder reaction performed with thiocarbonyl *S*-methanides and thiochalcones, respectively [37–38].

## Results and Discussion

In a preliminary experiment, the reaction of 1,4-naphthoquinone (**1a**) with *N*-phenyltrifluoroacetohydrazonoyl bromide (**8a**), used as an easy-to-handle precursor of nitrile imine **7a**, was examined. The reaction was performed in selected aprotic organic solvents by using organic and inorganic bases, such as Et_3_N, K_2_CO_3_, and DBU. Along with the “traditional” reaction performed in solution, a mechanochemical approach using ball milling was also tested. The obtained results are collected and compared in Table 1. They show that the best results were achieved using dry THF as a solvent and K_2_CO_3_ as a base; under these conditions, the best conversion of the starting materials and the highest yield (93%) for the expected product **9a** were observed. Typically, for cycloadducts obtained from 1,4-naphthoquinone [38], the initially formed [3 + 2]-cycloadduct **10a** smoothly underwent air oxidation during the work-up to give pyrazole **9a** as the final product.

**Table 1:** Results of the [3 + 2]-cycloaddition of quinone **1a** with the fluorinated nitrile imine **7a** generated from **8a**.

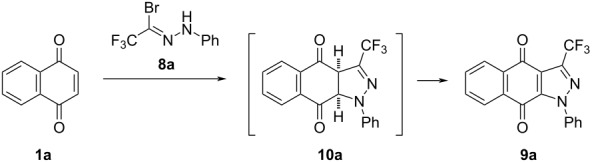			

base	solvent	reaction time	yield (%)^a^

Et_3_N	THF	2 d	93
K_2_CO_3_	THF	2 d	97 (93)
Cs_2_CO_3_	THF	2 d	90
DBU	THF	2 d	—^b^
K_2_CO_3_	DCM	2 d	96
K_2_CO_3_	toluene	2 d	22
K_2_CO_3_	—^c^	3 h	85
Et_3_N	—^c^	3 h	79^d^

^a^Conversion of naphthoquinone (**1a**) into **9a** estimated on the basis of ^1^H NMR spectra of the crude reaction mixture. Yield of isolated product given in parentheses. ^b^Pyrazole **9a** was not found in the mixture. ^c^Solvent-free ball mill reaction. ^d^Partial decomposition of the starting bromide **8a**.

It is worth mentioning that the solvent-free mechanochemical approach also led to the desired product, albeit a lower yield of **9a** was noticed. In these reactions, a sticky material was obtained, which was difficult to grind and therefore, completion of these reactions was practically impossible. On the other hand, the ball mill approach provided the target material after a significantly shorter reaction time (3 h instead of 2 d).

**Scheme 3:**
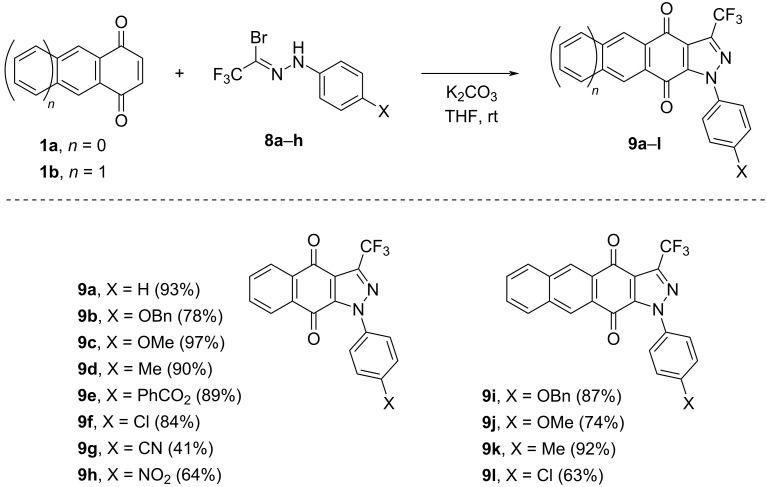
Synthesis of [3 + 2]-cycloadducts **9a**–**l** derived from CF_3_-substituted nitrile imines **7a**–**h** and 1,4-quinones **1a** and **1b**.

**Figure 2:**
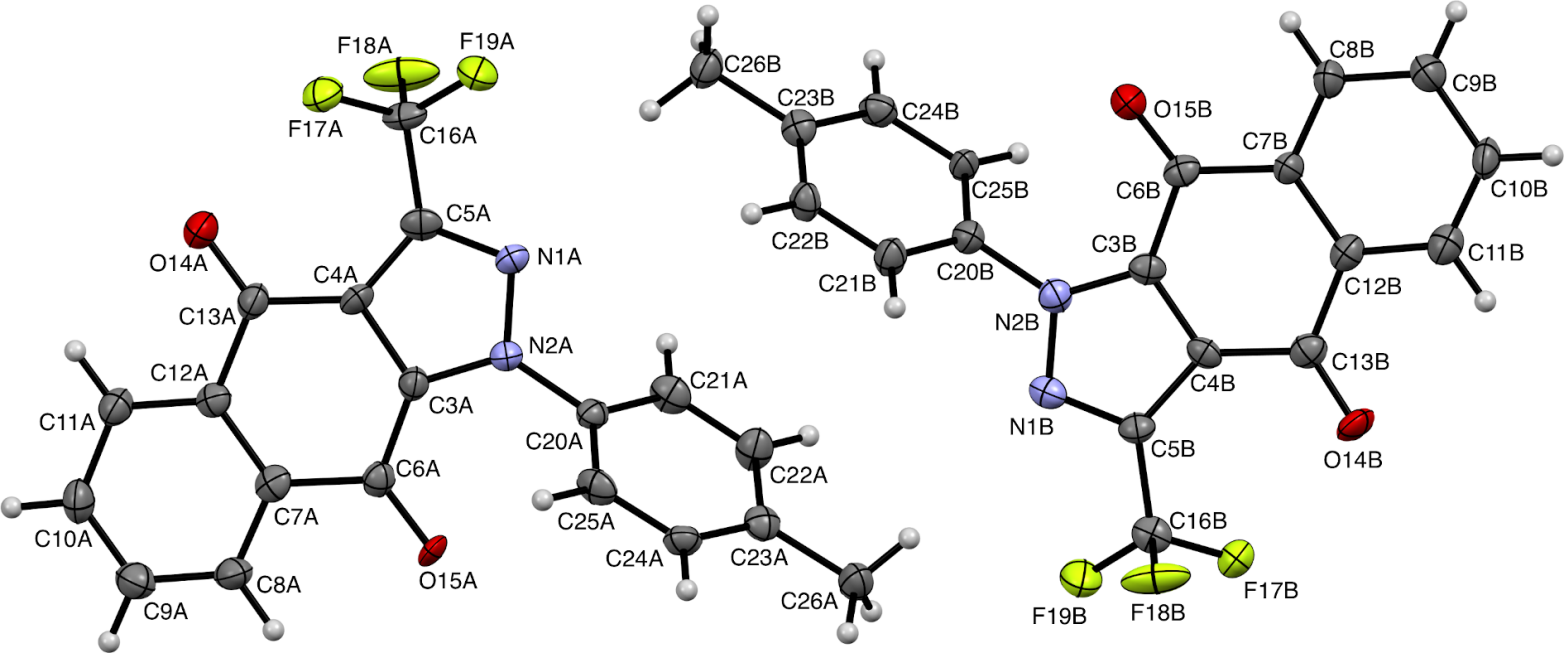
X-ray structure of 3-trifluoromethylpyrazole derivative **9d**. The labeling scheme of the asymmetric unit is shown. Anisotropic displacement parameters of nonhydrogen atoms are drawn as ellipsoids with a 50% probability level. N atoms in blue, O atoms in red, C atoms in grey, H atoms as small white spheres. Structure deposited under deposition number CCDC-2078498.

Based on the optimized protocol, a series of cycloadditions with nitrile imines **7** was carried out using **1a** (Scheme 3). Irrespective of the electronic properties of the substituent X located at the C(4) atom of the aryl ring in the precursor **8**, generally, a high yield of the isolated products **9** was observed (78–97%). Only for derivatives bearing strongly electron-withdrawing groups X (i.e., CN, NO_2_), a longer reaction time (up to 6 d) was required to complete these experiments, and the yield of the isolated products dropped significantly (to 41 and 64%, respectively). In analogy to the tricyclic pyrazole derivative **9a**, the expected products **9b**–**h** formed after spontaneous aromatization of the initial [3 + 2]-cycloadducts by air oxidation were isolated exclusively. Additionally, the X-ray structure of **9d** is shown in Figure 2.

Similar results were obtained starting with 1,4-anthraquinone (**1b**) and selected hydrazonoyl bromides **8**. In this series, fused pyrazoles **9i**–**l** were obtained in high yield (63–92%, Scheme 3).

**Scheme 4:**
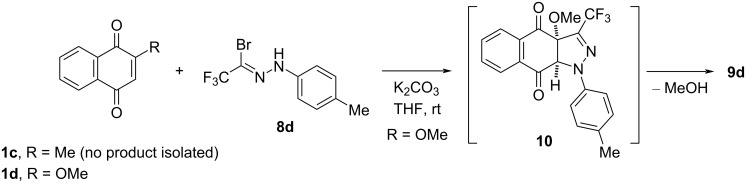
Thermal elimination of MeOH from the initial [3 + 2]-cycloadduct of **1d** and nitrile imine **7d** generated from **8d**.

Finally, the experiment performed under the optimized conditions with the nonsymmetric menadione (**1c**, vitamin K_3_), bearing the Me group at C(2), with in-situ-generated nitrile imines **7c** (X = OMe) and **7d** (X = Me) led to complex mixtures of unidentified products (Scheme 4). These results differ from that reported by Huisgen [17], who in the reaction of nitrile imine **2** with the same dipolarophile obtained the expected [3 + 2]-cycloadduct to the C=C bond as a single regioisomer, which was isolated in 33% yield. The observed outcome suggests that the thermally generated nitrile imine **2** undergoes [3 + 2]-cycloaddition more efficiently than fluorinated nitrile imines **7** generated under basic conditions. It seems likely that in the presence of a base, sequential reactions of the conjugated C–H-acidic quinone **1c** occur, which lead to a complex mixture of products. In contrast, the reaction of the same nitrile imine **7d** with the structurally analogous 1,4-quinone **1d**, bearing the OMe group at C(2), provided the known pyrazole **9d** as the only product in the excellent yield of 96% after typical aqueous work-up, although the reaction required a longer reaction time (3 d, Scheme 4). Presumably, the initially formed [3 + 2]-cycloadduct **10b** undergoes spontaneous elimination of MeOH, yielding the aromatized product **9d**. A similar mechanistic scenario was observed and discussed previously for the reactions of trifluoroacetonitrile imines **7** with enol ethers used as dipolarophiles [27].

**Scheme 5:**
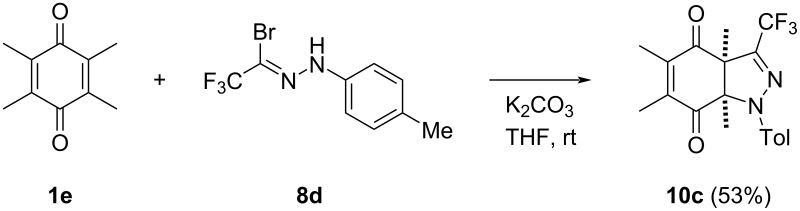
Formation of the thermally stable, initially formed [3 + 2]-cycloadduct **10c** obtained from **1e** and nitrile imine **7d** generated from **8d**.

An additional experiment deserves a brief comment. The [3 + 2]-cycloaddition of **7d** (X = Me), generated from **8d**, with 2,3,5,6-tetramethyl-1,4-benzoquinone (**1e**), performed under the optimized conditions (2 d at rt), yielded the fairly stable nonaromatic [3 + 2]-cycloadduct **10c**, which was isolated by chromatographic work-up in 53% yield (Scheme 5).

The presence of ≈40% of unconsumed quinone **1e** in the crude reaction mixture evidenced that the observed fully chemoselective cycloaddition reaction with the sterically congested C=C bond in **1e** occurs less efficiently than in 2,3-unsubstituted 1,4-quinones, such as **1a** and **1b**. However, in that case, the subsequent elimination step leading to an aromatized product cannot take place.

**Figure 3:**
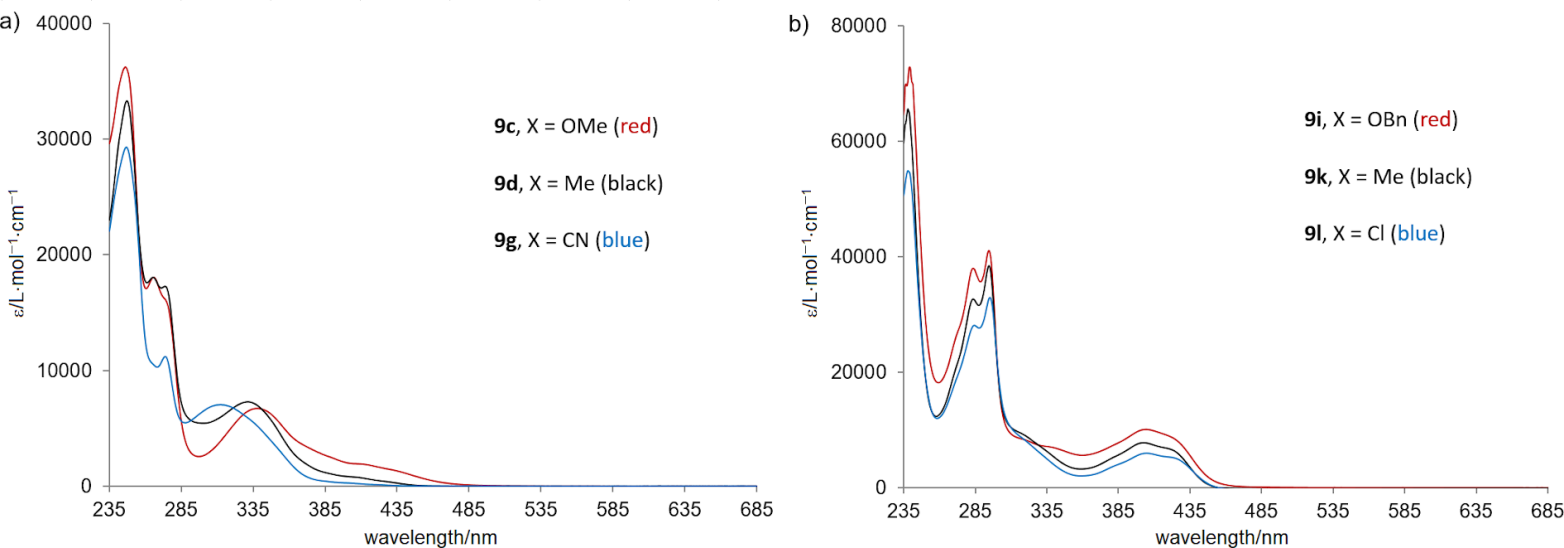
Electronic absorption spectra for selected a) naphthoquinone-derived (**9c**, **9d**, **9g**) and b) anthraquinone-derived (**9i**, **9k**, **9l**) trifluoromethylated pyrazoles in CH_2_Cl_2_.

All products of the type **9** are colored, typically pale yellow, both in the solid state and in solution. The UV–vis spectroscopic analysis of the naphthoquinone-derived series (compounds **9a**–**h**) revealed less intense absorption in the visible range at ≈410 nm and medium-intensity absorption between 310–340 nm. In the latter region, a significant hypsochromic shift of the maxima with increasing electron-withdrawing character of the substituent X could be observed (Figure 3).

Remarkably, the 1,4-anthraquinone-derived products **9i**, **9k**, and **9l** exhibit two overlapping absorption bands in the visible range with maxima at λ_max_ ≈ 400 nm and ≈ 425 nm, respectively.

## Conclusion

The presented study demonstrated that 1,4-quinones undergo efficient [3 + 2]-cycloadditions with in-situ-generated electron-deficient trifluoroacetonitrile imines to give polycyclic pyrazole derivatives as exclusive products formed via spontaneous air oxidation of the initial [3 + 2]-cycloadducts. In contrast to some arylnitrile oxides and benzonitrile ylides, no competitive cycloaddition to the C=O bonds of the quinone molecule has been observed, and all studied reactions proceeded chemoselectively with the C=C bond as the only dipolarophilic center. This result corresponds to other reported cases in which nonfluorinated nitrile imines were employed to form fused pyrazoles in reactions with 1,4-quinones. Interestingly, reactions of *para*-quinone methides with common nitrile imines proceeded with different chemoselectivity, and in these cases, the exocyclic C=C bond played the role of the exclusive dipolarophilic center, yielding corresponding spiropyrazoles [39–40].

This unusual selectivity in [3 + 2]-cycloadditions of less electron-deficient nitrile imines was studied computationally to rationalize the observed pathways [41]. Hence, it is worth mentioning that the fused pyrazoles, which are attractive compounds for diverse practical applications in medicinal and materials chemistry, are available not only by the aza-Nenitzescu reaction [42–43] but also via simple and highly efficient [3 + 2]-cycloadditions of nitrile imines with the C=C bond of 1,4-quinones. In general, the present study emphasizes the great importance of nitrile imines as versatile 1,3-dipoles useful for the preparation of N-heterocycles of potential importance for medicinal chemistry, agrochemistry, and materials chemistry [44–45].

## Experimental

### General information

If not stated otherwise, reactions were carried out under inert atmosphere (argon) in flame-dried flasks with addition of the reactants by using syringes; subsequent manipulations were conducted in air. Products were purified by standard column chromatography (CC) on silica gel (230–400 mesh; deactivated prior to use with 2% Et_3_N in petroleum ether) by using freshly distilled solvents as eluents (petroleum ether, CH_2_Cl_2_, AcOEt) or recrystallized from hot CHCl_3_. THF was dried over sodium and benzophenone and freshly distilled before use; anhydrous DMF was purchased and used as received. The NMR spectra were measured on a Bruker AVIII instrument (^1^H at 600 MHz, ^13^C at 151 MHz, and ^19^F at 565 MHz). Chemical shifts are reported relative to residual undeuterated solvent peaks (for CDCl_3_: ^1^H NMR δ = 7.26 ppm, ^13^C NMR δ = 77.0 ppm; for 1,1,2,2-tetrachloroethane-*d*_2_ (C_2_D_2_Cl_4_): ^1^H NMR δ = 6.0 ppm, ^13^C NMR δ = 73.8 ppm) or to CFCl_3_ (^19^F NMR δ = 0.00 ppm) used as external standard. Multiplicities of the signals in ^13^C NMR spectra were assigned based on supplementary 2D measurements (COSY, HMQC, and HMBC). The UV–vis spectra were measured on a PerkinElmer Lambda 45 spectrophotometer in spectroscopic grade CH_2_Cl_2_. MS (ESI) were performed with a Varian 500-MS LC Ion Trap. The IR spectra were measured neat with an Agilent Cary 630 FTIR spectrometer. Elemental analyses were obtained with a Vario EL III instrument (Elementar Analysensysteme GmbH). Melting points were determined in capillaries with a MEL-TEMP apparatus (Aldrich) or with a polarizing optical microscope (Opta-Tech) and are uncorrected.

### Starting materials

The requisite hydrazonoyl bromides **8a**–**h** were prepared by NBS-mediated bromination of the corresponding trifluoroacetaldehyde hydrazones in dry DMF at room temperature as described in an earlier publication [21]. The latter arylhydrazones were obtained according to a general literature protocol by condensation of aqueous fluoral hydrate (≈75% in H_2_O) with commercially available hydrazines in a closed ampoule at 75 °C in methanol in the presence of molecular sieves (4 Å) [46].

### General procedure

To a stirred solution of the respective 1,4-quinone **1** (1.0 mmol) and K_2_CO_3_ in dry THF (10 mL), a hydrazonoyl bromide **8** (1.1 mmol) was added, and stirring was continued at room temperature until the starting material **1** was fully consumed (based on TLC monitoring, petroleum ether/dichloromethane 1:1). After the resulting precipitate and unconsumed carbonate were filtered off, the solvent was removed under reduced pressure. The crude mixtures were purified by CC using silica gel as the stationary phase and either petroleum ether (or hexanes)/dichloromethane or petroleum ether/AcOEt mixtures as eluent to give analytically pure products **9a**–**h** and **10c**.

#### 1-Phenyl-3-(trifluoromethyl)-1*H*-benzo[*f*]indazole-4,9-dione (**9a**)

Reaction time 2 d; CC (SiO_2_, petroleum ether/CH_2_Cl_2_ 2:1); 320 mg (93%); yellow solid; mp 196–197 °C; ^1^H NMR (CDCl_3_, 600 MHz) δ 7.56–7.62 (m, 5H, Ph), 7.79, 7.84 (2 t, *J* = 7.5 Hz, 1H each, C_6_H_4_), 8.17, 8.30 (2 d, *J* = 7.7 Hz, 1H each, C_6_H_4_); ^13^C NMR (CDCl_3_, 151 MHz) δ 119.9 (q, ^1^*J*_C,F_ = 270.4 Hz, CF_3_), 120.8 (*i*-C), 125.7 (2CH), 127.3, 127.5 (CH each), 129.0 (2CH), 130.3 (CH), 132.9, 133.4 (2*i*-C), 134.2, 135.0 (CH each), 138.1, 139.0 (2*i*-C), 140.8 (q, ^2^*J*_C,F_ = 40.8 Hz, C(3)), 174.5, 177.3 (2C=O); ^19^F NMR (CDCl_3_, 565 MHz) δ −62.82 ppm; UV–vis (CH_2_Cl_2_) λ_max_ (log ε) 247 (4.45), 266 (4.24), 275 (4.25), 340 (3.72), 409 (2.61), 496 nm (1.70); IR (neat) ν_max_: 3082, 1677 (C=O), 1588, 1521, 1495, 1331, 1279, 1230, 1133, 1100, 921, 716 cm^−1^; ESIMS (*m*/*z*): 365.1 (100, [M + Na]^+^), 343.1 (12, [M + H]^+^); Anal. calcd for C_18_H_9_F_3_N_2_O_2_: C, 63.16; H, 2.65; N, 8.18; found: C, 63.34; H, 2.63; N, 8.24 (all values are given as percentages).

#### 3a,5,6,7a-Tetramethyl-1-(*p*-tolyl)-3-(trifluoromethyl)-3a,7a-dihydro-1*H*-indazole-4,7-dione (**10c**)

Reaction time 2 d; CC (SiO_2_, petroleum ether/AcOEt 20:1); 193 mg (53%); yellow oil; ^1^H NMR (CDCl_3_, 600 MHz) δ 1.39 (s, 3H, Me), 1.52 (q_br_, *J* = 0.5 Hz, 3H, Me), 2.05–2.07 (m, 6H, 2 Me), 2.32 (s, 3H, Me), 6.95–6.97, 7.10–7.12 (2 m, 2H each, Tol); ^13^C NMR (CDCl_3_, 151 MHz) δ 13.6, 13.9, 14.2, 14.6, 20.8 (5Me), 64.8, 80.1 (2*i*-C), 120.7 (q,^1^*J*_C,F_ = 271.1 Hz, CF_3_), 120.7, 129.6 (2CH each), 134.8 (*i*-C), 140.0 (q, ^2^*J*_C,F_ = 36.3 Hz, C(3)), 139.5, 146.9, 147.1 (3*i*-C), 192.6, 195.1 (2C=O) ppm; ^19^F NMR (CDCl_3_, 565 MHz) δ −61.45 ppm; IR (neat) ν_max_: 2930, 1677 (C=O), 1513, 1506, 1379, 1267, 1170, 1118, 1070, 1029, 954, 850, 816, 712 cm^−1^; ESIMS (*m*/*z*): 365.4 (100, [M + H]^+^); Anal. calcd for C_19_H_19_F_3_N_2_O_2_: C, 62.63; H, 5.26; N, 7.69; found: C, 62.76; H, 5.39; N, 7.95.

## Supporting Information

File 1 General information and experimental data of all isolated products, details of the crystal structure determination, and copies of ^1^H and ^13^C NMR spectra for all products.

## References

[1] El-Najjar N, Gali-Muhtasib H, Ketola R A, Vuorela P, Urtti A, Vuorela H (2011). Phytochem Rev.

[2] Wang Y, Zhu S, Zou L-H (2019). Eur J Org Chem.

[3] Han C, Li H, Shi R, Zhang T, Tong J, Li J, Li B (2019). J Mater Chem A.

[4] Patel O P S, Beteck R M, Legoabe L J (2021). Eur J Med Chem.

[5] Nawrat C C, Moody C J (2014). Angew Chem, Int Ed.

[6] Tapia R A, Carrasco C, Ojeda S, Salas C, Valderrama J A, Morello A, Repetto Y (2002). J Heterocycl Chem.

[7] Wang C, Chen X-H, Zhou S-M, Gong L-Z (2010). Chem Commun.

[8] Berhe S, Slupe A, Luster C, Charlier H A, Warner D L, Zalkow L H, Burgess E M, Enwerem N M, Bakare O (2010). Bioorg Med Chem.

[9] Huang H-M, Gao J-R, Ye Q, Yu W-B, Sheng W-J, Li Y-J (2014). RSC Adv.

[10] Morrocchi S, Quilico A, Ricca A, Selva A (1968). Gazz Chim Ital.

[11] Shiraishi S, Holla B S, Imamura K (1983). Bull Chem Soc Jpn.

[12] Hamadi N B, Msaddek M (2006). Heterocycl Commun.

[13] Shiraishi S, Ikeuchi S, Senō M, Asahara T (1977). Bull Chem Soc Jpn.

[14] Shiraishi S, Ikeuchi S, Senō M, Asahara T (1978). Bull Chem Soc Jpn.

[15] Stegmann W, Uebelhart P, Heimgartner H (1983). Helv Chim Acta.

[16] Gilgen P, Jackson B, Hansen H-J, Heimgartner H, Schmid H (1974). Helv Chim Acta.

[17] Huisgen R, Seidel M, Wallbillich G, Knupfer H (1962). Tetrahedron.

[18] Argyropoulos N G, Mentzafos D, Terzis A (1990). J Heterocycl Chem.

[19] Ortiz‐Rojano L, Rojas‐Martín J, Rodríguez‐Diaz C, Carreño M C, Ribagorda M (2019). Chem – Eur J.

[20] Bertuzzi G, Crotti S, Calandro P, Bonini B F, Monaco I, Locatelli E, Fochi M, Zani P, Strocchi E, Mazzanti A (2018). ChemMedChem.

[21] Mlostoń G, Urbaniak K, Utecht G, Lentz D, Jasiński M (2016). J Fluorine Chem.

[22] Utecht G, Sioma J, Jasiński M, Mlostoń G (2017). J Fluorine Chem.

[23] Grzelak P, Utecht G, Jasiński M, Mlostoń G (2017). Synthesis.

[24] Utecht-Jarzyńska G, Jasiński M, Świątek K, Mlostoń G, Heimgartner H (2020). Heterocycles.

[25] Utecht-Jarzyńska G, Mykhaylychenko S S, Rusanov E B, Shermolovich Y G, Jasiński M, Mlostoń G (2021). J Fluorine Chem.

[26] Utecht-Jarzyńska G, Michalak A, Banaś J, Mlostoń G, Jasiński M (2019). J Fluorine Chem.

[27] Utecht G, Fruziński A, Jasiński M (2018). Org Biomol Chem.

[28] Utecht G, Mlostoń G, Jasiński M (2018). Synlett.

[29] Tian Y-C, Li J-K, Zhang F-G, Ma J-A (2021). Adv Synth Catal.

[30] Kowalczyk A, Utecht-Jarzyńska G, Mlostoń G, Jasiński M (2021). J Fluorine Chem.

[31] Fustero S, Sánchez-Roselló M, Barrio P, Simón-Fuentes A (2011). Chem Rev.

[32] Kaur K, Kumar V, Gupta G K (2015). J Fluorine Chem.

[33] Zhou Y, Wang J, Gu Z, Wang S, Zhu W, Aceña J L, Soloshonok V A, Izawa K, Liu H (2016). Chem Rev.

[34] Hu X-G, Hunter L (2013). Beilstein J Org Chem.

[35] Mykhailiuk P K (2021). Chem Rev.

[36] Lipunova G N, Nosova E V, Charushin V N, Chupakhin O N (2015). J Fluorine Chem.

[37] Mlostoń G, Urbaniak K, Urbaniak P, Marko A, Linden A, Heimgartner H (2018). Beilstein J Org Chem.

[38] Mlostoń G, Celeda M, Heimgartner H (2003). Heterocycles.

[39] Woolhouse A D (1977). Aust J Chem.

[40] Su Y, Zhao Y, Chang B, Zhao X, Zhang R, Liu X, Huang D, Wang K-H, Huo C, Hu Y (2019). J Org Chem.

[41] Soleymani M, Jahanparvar S (2020). Monatsh Chem.

[42] Lyubchanskaya V M, Alekseeva L M, Granik V G (1999). Chem Heterocycl Compd.

[43] Janardhanan J C, Mishra R K, Das G, Sini S, Jayamurthy P, Suresh C H, Praveen V K, Manoj N, Babu B P (2018). Asian J Org Chem.

[44] Quadrelli P (2019). Modern Applications of Cycloaddition Chemistry.

[45] Jamieson C, Livingstone K (2020). The Nitrile Imine 1,3-Dipoles – Properties, Reactivity and Applications.

[46] Wojciechowska A, Jasiński M, Kaszyński P (2015). Tetrahedron.

