# Supplementary material for: A straightforward conversion of 1,4-quinones into polycyclic pyrazoles via [3 + 2]-cycloaddition with fluorinated nitrile imines
Source: Beilstein J Org Chem. 2021 Jun 28;17:1509–17. doi: 10.3762/bjoc.17.108 (PMC8261526; doi:10.3762/bjoc.17.108)
Supplement: File 1 — General information and experimental data of all isolated products, details of the crystal structure determination, and copies of 1H and 13C NMR spectra for all products. [file Beilstein_J_Org_Chem-17-1509-s001.pdf]

BEILSTEIN JOURNAL OF ORGANIC CHEMISTRY

Supporting Information

for

A straightforward conversion of 1,4-quinones into polycyclic pyrazoles via [3 + 2]-cycloaddition with fluorinated nitrile imines

Greta Utecht-Jarzyńska, Karolina Nagła, Grzegorz Mlostoń, Heinz Heimgartner, Marcin Palusiak and Marcin Jasiński

Beilstein J. Org. Chem. **2021,** *17,* 1509–1517. doi:10.3762/bjoc.17.108

General information and experimental data of all isolated products, details of the crystal structure determination, and copies of ^{1}H and ^{13}C NMR spectra for all products

License and Terms: This is a supporting information file under the terms of the Creative Commons Attribution License (https://creativecommons.org/licenses/by/4.0). Please note that the reuse, redistribution and reproduction in particular requires that the author(s) and source are credited and that individual graphics may be subject to special legal provisions.
The license is subject to the *Beilstein Journal of Organic Chemistry* terms and conditions: (https://www.beilstein-journals.org/bjoc/terms)

Table of contents

Experimental data of all synthesized compounds S2

Copies of ^{1}H and ^{13}C NMR spectra S13

UV–Vis measurements S39

X-ray data of pyrazole **9d** S41

Experimental data of all synthesized compounds

General procedure: To a stirred solution of the respective quinone **1** (1.0 mmol) and K_2CO_3 in dry THF (10 mL), a hydrazonoyl bromide **8** (1.1 mmol) was added, and the stirring was continued at room temperature until the starting quinone was fully consumed (based on TLC monitoring, petroleum ether/dichloromethane 1:1). After the resulting precipitate and unconsumed carbonate were filtered off, the solvent was removed under reduced pressure. The resulting mixture was purified by column chromatography (CC) using SiO_2 as the stationary phase and petroleum ether (or hexanes)/dichloromethane mixtures as an eluent to give analytically pure products **9a**–**9h** and **10c**.

1-(4-Benzyloxyphenyl)-3-(trifluoromethyl)-1*H*-benzo[*f*]indazole-4,9-dione (9b)

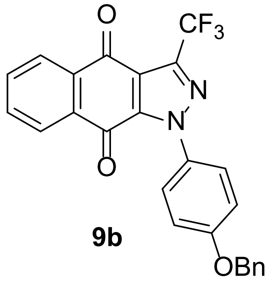

9b

Reaction time: 2 d; CC (SiO_2, petroleum ether/CH_2Cl_2 1:1), 350 mg (78%), yellow solid, mp 209–210 °C. ^{1}H NMR (600 MHz, $CDCl_3$): δ 5.16 (s, 2 H, CH_2), 7.13 (d_{br}, J = 9.0 Hz, 2 H, C_6H_4), 7.37, 7.42 (2 t_{br}, J ≈ 7.3 Hz, 1 H, 2 H, Ph), 7.47 (d_{br}, J ≈ 7.3 Hz, 2 H, Ph), 7.53 (d_{br}, J ≈ 9.0 Hz, 2H, C_6H_4), 7.79, 7.84 (2 td, J = 7.5, 1.3 Hz, 1 H each, C_6H_4), 8.17, 8.30 (2 dd, J = 7.7, 1.1 Hz, 1 H each, C_6H_4) ppm; ^{13}C NMR (151 MHz, $CDCl_3$): δ 70.4 (CH_2), 115.0 (2 CH), 119.9 (q, $^1J_{C,F}$ = 270.4 Hz, CF_3), 120.6 (*i*-C), 127.0 (2 CH), 127.3, 127.5 (CH each), 127.5 (2 CH), 128.3 (CH), 128.7 (2 CH), 131.2, 133.0, 133.5 (3 *i*-C), 134.1, 134.9 (CH each), 136.2, 138.9 (2 *i*-C), 140.5 (q, $^2J_{C,F}$ = 40.7

Hz, C-3), 160.0 (*i*-C), 174.6, 177.4 (2 C=O) ppm; ^{19}F NMR (565 MHz, $CDCl_3$): δ –62.80 ppm; UV (CH_2Cl_2), λ_{max} (log ε) 247 (4.59), 265 (4.28), 279 (4.16), 344 (3.83), 417 (3.30), 442 (3.08); IR (neat): ν 2922, 1677 (C=O), 1588, 1506, 1334, 1290, 1230, 1178, 1144, 1111, 1003, 921, 835, 749 cm^{-1}; ESI-MS (*m*/*z*): 449.4 (25, $[M + H]^+$), 471.4 (100, $[M + Na]^+$), 487.3 (12, $[M + K]^+$); elemental analysis calcd (%) for $C_{25}H_{15}F_3N_2O_3$ (448.4): C 66.97, H 3.37, N 6.25; found: C 67.12, H 3.54, N 6.35.

1-(4-Methoxyphenyl)-3-(trifluoromethyl)-1*H*-benzo[*f*]indazole-4,9-dione (**9c**)

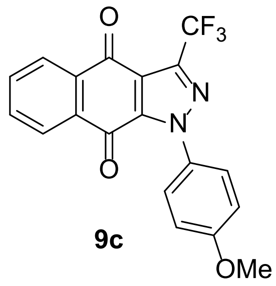

9c

Reaction time: 2 d; CC (SiO_2, petroleum ether/CH_2Cl_2 1:1), 361 mg (97%), yellow solid, mp 217–218 °C. ^{1}H NMR (600 MHz, $CDCl_3$): δ 3.91 (s, 3 H, OMe), 7.06, 7.53 (2 d_{br}, J = 8.9 Hz, 2 H each, C_6H_4), 7.78, 7.84 (2 td, J = 7.5, 1.2 Hz, 1 H each, C_6H_4), 8.17, 8.30 (2 dd, J = 7.7, 1.0 Hz, 1 H each, C_6H_4) ppm; ^{13}C NMR (151 MHz, $CDCl_3$): δ 55.7 (OMe), 114.2 (2 CH), 120.0 (q, $^1J_{C,F}$ = 270.7 Hz, CF_3), 120.6 (*i*-C), 127.0 (2 CH), 127.3, 127.5 (CH each), 131.0, 133.0, 133.5 (3 *i*-C), 134.1, 134.9 (CH each), 138.9 (*i*-C), 140.5 (q, $^2J_{C,F}$ = 40.4 Hz, C-3), 160.9 (*i*-C), 174.6, 177.4 (2 C=O) ppm; ^{19}F NMR (565 MHz, $CDCl_3$): δ –62.80 ppm; UV (CH_2Cl_2), λ_{max} (log ε) 253 (4.44), 273 (4.21), 280 (4.06), 345 (3.81), 419 (3.24), 446 (2.96); IR (neat): ν 3323, 1685 (C=O), 1588, 1506, 1290, 1256, 1144, 1111, 1025, 924, 835, 719 cm^{-1}; ESI-MS (*m*/*z*): 395.3 (100, $[M + Na]^+$);

elemental analysis calcd (%) for $C_{19}H_{11}F_3N_2O_3$ (372.3): C 61.30, H 2.98, N 7.52; found: C 61.31, H 3.07, N 7.78.

1-(*p*-Tolyl)-3-(trifluoromethyl)-1*H*-benzo[*f*]indazole-4,9-dione (**9d**)

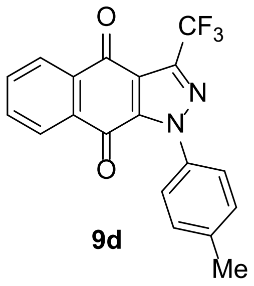

9d

Reaction time: 2 d; CC (SiO_2, petroleum ether/CH_2Cl_2 2:1), 321 mg (90%), pale yellow solid, mp 246–247 °C. ^{1}H NMR (600 MHz, $CDCl_3$): δ 2.48 (s, 3 H, Me), 7.37, 7.48 (2 d, $J \approx 8.2$ Hz, 2 H each, Tol), 7.78, 7.84 (2 td, J = 7.5, 1.3 Hz, 1 H each, C_6H_4), 8.16, 8.30 (2 dd, $J \approx 7.7$, 1.1 Hz, 1 H each, C_6H_4) ppm; ^{13}C NMR (151 MHz, $CDCl_3$): δ 21.4 (Me), 119.9 (q, $^1J_{C,F}$ = 270.4 Hz, CF_3), 120.1 (*i*-C), 125.5 (2 CH), 127.3, 127.5 (CH each), 129.6 (2 CH), 133.0, 134.2 (2 *i*-C), 134.2, 134.9 (CH each), 135.7, 139.0 (2 *i*-C), 140.6 (q, $^2J_{C,F}$ = 40.7 Hz, C-3), 140.7 (*i*-C), 174.5, 177.4 (2 C=O) ppm; ^{19}F NMR (565 MHz, $CDCl_3$): δ –62.80 ppm; UV (CH_2Cl_2), λ_{max} (log ε) 253 (4.43), 272 (4.23), 279 (4.15), 339 (3.84), 414 (2.80), 434 (2.46); IR (neat): ν 3343, 1681 (C=O), 1584, 1506, 1331, 1286, 1234, 1178, 1129, 1103, 917, 824, 716 cm^{-1}; ESI-MS (*m*/*z*): 379.3 (100, [M + Na]$^+$); elemental analysis calcd (%) for $C_{19}H_{11}F_3N_2O_2$ (356.3): C 64.05, H 3.11, N 7.86; found: C 64.06, H 3.12, N 8.00.

4-(4,9-Dioxo-3-(trifluoromethyl)-4,9-dihydro-1*H*-benzo[*f*]indazol-1-yl)phenyl benzoate (9e)

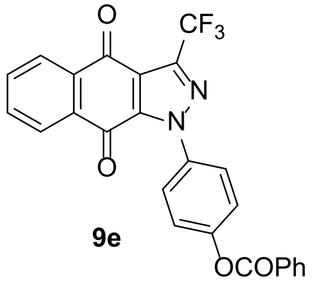

9e

Reaction time: 2 d; CC (SiO_2, petroleum ether/CH_2Cl_2 1:1), 412 mg (89%), yellow solid, mp 251–252 °C. ^{1}H NMR (600 MHz, $CDCl_3$): δ 7.46 (d_{br}, J = 8.9 Hz, 2 H, C_6H_4), 7.55 (t_{br}, J = 7.8 Hz, 2 H, Ph), 7.68 (t_{br}, J = 7.4 Hz, 1 H, Ph), 7.71 (d_{br}, J = 8.0 Hz, 2 H, C_6H_4), 7.80, 7.85 (2 td, J = 7.5, 1.3 Hz, 1 H each, C_6H_4), 8.19 (dd, J ≈ 7.7, 1.1 Hz, 1 H, C_6H_4), 8.24 (d_{br}, J = 8.3 Hz, 2 H, Ph), 8.31 (dd, J = 7.7, 1.1 Hz, 1 H, C_6H_4) ppm; ^{13}C NMR (151 MHz, $CDCl_3$): δ 119.9 (q, $^1J_{C,F}$ = 270.5 Hz, CF_3), 120.9 (*i*-C), 122.4, 127.0 (2 CH each), 127.4, 127.5 (CH each), 128.7 (2 CH), 129.0 (*i*-C), 130.3 (2 CH), 132.9, 133.4 (2 *i*-C), 133.9, 134.2, 135.0 (3 CH each), 135.5, 139.1 (2 *i*-C), 140.9 (q, $^2J_{C,F}$ = 40.6 Hz, C-3), 152.1 (*i*-C), 164.6, 174.5, 177.3 (3 C=O) ppm; ^{19}F NMR (565 MHz, $CDCl_3$): δ –62.85 ppm; UV (CH_2Cl_2), λ_{max} (log ε) 251 (4.53), 271 (4.17), 279 (4.05), 336 (3.82), 412 (2.51); IR (neat): ν 2922, 1733 (C=O), 1681 (C=O), 1588, 1506, 1271, 1219, 1129, 1062, 917, 708 cm^{-1}; ESI-MS (*m*/*z*): 485.3 (55, $[M + Na]^+$), 501.3 (100, $[M + K]^+$); elemental analysis calcd (%) for $C_{25}H_{13}F_3N_2O_4$ (462.4): C 64.94, H 2.83, N 6.06; found: C 64.85, H 3.08, N 6.02.

1-(4-Chlorophenyl)-3-(trifluoromethyl)-1*H*-benzo[*f*]indazole-4,9-dione (**9f**)

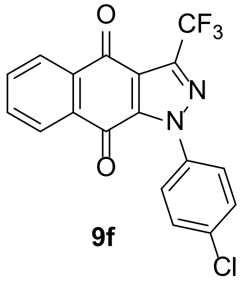

9f

Reaction time: 2 d; CC (SiO_2, petroleum ether/CH_2Cl_2 2:1), 316 mg (84%), yellow solid, mp 213–214 °C. ^{1}H NMR (600 MHz, $CDCl_3$): δ 7.55, 7.58 (2 d_{br}, J = 8.9 Hz, 2 H each, C_6H_4), 7.80, 7.85 (2 td, J = 7.5, 1.3 Hz, 1 H each, C_6H_4), 8.17, 8.30 (2 dd, J = 7.7, 1.0 Hz, 1 H each, C_6H_4) ppm; ^{13}C NMR (151 MHz, $CDCl_3$): δ 119.8 (q, $^1J_{C,F}$ = 270.5 Hz, CF_3), 121.0 (*i*-C), 127.0 (2 CH), 127.4, 127.6 (CH each), 129.3 (2 CH), 132.8, 133.3 (2 *i*-C), 134.3, 135.1 (CH each), 136.4, 136.5, 139.0 (3 *i*-C), 141.0 (q, $^2J_{C,F}$ = 40.9 Hz, C-3), 174.5, 177.2 (2 C=O) ppm; ^{19}F NMR (565 MHz, $CDCl_3$): δ –62.88 ppm; UV (CH_2Cl_2), λ_{max} (log ε) 254 (4.35), 272 (4.16), 279 (4.07), 337 (3.79), 393 (2.80), 411 (2.61), 432 (2.20); IR (neat): ν 3071, 1677 (C=O), 1592, 1491, 1290, 1234, 1182, 1129, 921, 835, 738 cm^{-1}; ESI-MS (*m*/*z*): 377.3 (10, $[M + H]^+$), 399.2 (100, $[M + Na]^+$); elemental analysis calcd (%) for $C_{18}H_8ClF_3N_2O_2$ (376.7): C 57.39, H 2.14, N 7.44; found: C 57.37, H 2.25, N 7.66.

4-(4,9-Dioxo-3-(trifluoromethyl)-4,9-dihydro-1*H*-benzo[f]indazol-1-yl)benzonitrile (9g)

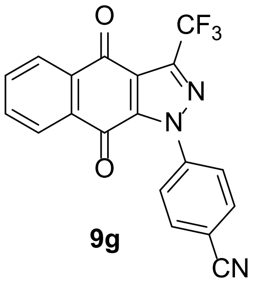

9g

Reaction time: 6 d; CC (SiO_2, petroleum ether/CH_2Cl_2 1:1 gradient CH_2Cl_2), 151 mg (41%), pale yellow solid, mp 247–248 °C. ^{1}H NMR (600 MHz, $CDCl_3$): δ 7.81–7.84, 7.86–7.89 (2 m, 3 H each), 8.19, 8.31 (2 dd, *J* = 7.6, 0.9 Hz, 1 H each, C_6H_4) ppm; ^{13}C NMR (151 MHz, $CDCl_3$): δ 114.2 (CN), 117.6 (*i*-C), 119.7 (q, $^1J_{C,F}$ = 270.6 Hz, CF_3), 121.5 (*i*-C), 126.6 (2 CH), 127.5, 127.7 (CH each), 132.7 (*i*-C), 133.0 (2 CH), 133.2 (*i*-C), 134.4, 135.4 (CH each), 139.3, 141.2 (2 *i*-C), 141.7 (q, $^2J_{C,F}$ = 40.8 Hz, C-3), 174.5, 177.0 (2 C=O) ppm; ^{19}F NMR (565 MHz, $CDCl_3$): δ –62.96 ppm; UV (CH_2Cl_2), λ_{max} (log ε) 252 (4.41), 278 (3.96), 320 (3.83), 391 (2.52), 408 (2.28), 427 (1.82); IR (neat): ν 2922, 2229 (CN), 1674 (C=O), 1584, 1498, 1398, 1331, 1282, 1234, 1181, 1137, 1107, 921, 846, 716 cm^{-1}; ESI-MS (*m*/*z*): 368.1 (10, [M + H]$^+$), 390.2 (100, [M + Na]$^+$); elemental analysis calcd (%) for $C_{19}H_8F_3N_3O_2$ (367.3): C 62.13, H 2.20, N 11.44; found: C 62.13, H 2.40, N 11.36.

1-(4-Nitrophenyl)-3-(trifluoromethyl)-1*H*-benzo[*f*]indazole-4,9-dione (9h)

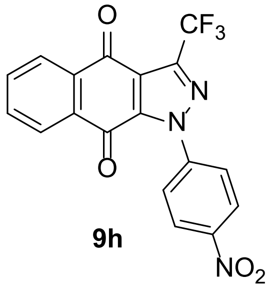

9h

Reaction time: 3 d; CC (SiO_2, hexanes/CH_2Cl_2 2:1), 245 mg (64%), yellow solid, mp 219–220 °C. ^1H NMR (600 MHz, $CDCl_3$): δ 7.83 (td, J = 7.5, 1.3 Hz, 1 H, C_6H_4), 7.87–7.91 (m, 3 H, C_6H_4), 8.20, 8.32 (2 dd, J = 7.7, 1.0 Hz, 1 H each, C_6H_4), 8.45 (d_{br}, J = 9.0 Hz, 2 H, C_6H_4) ppm; ^{13}C NMR (151 MHz, $CDCl_3$): δ 119.6 (q, $^1J_{C,F}$ = 270.7 Hz, CF_3), 121.6 (*i*-C), 124.4, 126.8 (2 CH each), 127.5, 127.7 (CH each), 132.7, 133.2 (2 *i*-C), 134.5, 135.4 (CH each), 139.4 (*i*-C), 141.8 (q, $^2J_{C,F}$ = 41.2 Hz, C-3), 142.5, 148.3 (2 *i*-C), 174.5, 177.0 (2 C=O) ppm; ^{19}F NMR (565 MHz, $CDCl_3$): δ –62.97 ppm; UV (CH_2Cl_2), λ_{max} (log ε) 253 (4.41), 259 (4.32), 279 (4.17), 312 (4.03), 356 (3.64), 388 (2.89), 408 (2.38), 425 (1.83); IR (neat): ν 2922, 2851, 1677 (C=O), 1587, 1528, 1495, 1349, 1286, 1234, 1141, 1103, 921, 857, 704 cm^{-1}; ESI-MS (*m*/*z*): 410.3 (100, $[M + Na]^+$); elemental analysis calcd (%) for $C_{18}H_8F_3N_3O_4$ (387.3): C 55.83, H 2.08, N 10.85; found: C 55.87, H 2.31, N 10.87.

Synthesis of pyrazoles 9i–9l: Following the general procedure, to a stirred solution of 1,4-anthraquinone (**1b**, 1.0 mmol, 208 mg) and K_2CO_3 (2.2 mmol, 304 mg) in dry THF (10 mL) hydrazonoyl bromide **8** (1.1 mmol) was added, and the stirring was continued at room temperature for 2 d. After H_2O (15 mL) was added, the mixture was extracted with DCM

(3×15 mL), the combined organic layers were dried over Na_2SO_4, and the solvents were removed in vacuo. The resulting products were recrystallized from hot $CHCl_3$. Due to low concentration of the samples (limited solubility of the products **9i**–**9l** in common organic solvents) and due to partial overlap of the signals in the ^{13}C NMR spectra, some of the low-intensity absorptions of the *C*F_3 and *C*-CF_3 atoms could not be found/interpreted.

1-(4-Benzyloxyphenyl)-3-(trifluoromethyl)-1*H*-naphtho[2,3-*f*]indazole-4,11-dione (9i)

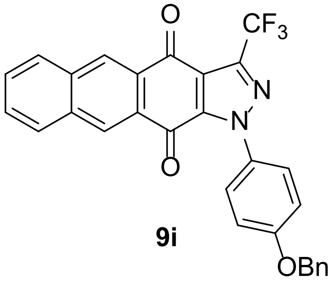

9i

Reaction time: 2 d, 434 mg (87%), yellow solid, mp 274–275 °C. ^{1}H NMR (600 MHz, $CDCl_3$): δ 5.18 (s, 2 H, CH_2), 7.14 (d_{br}, *J* = 8.9 Hz, 2 H, C_6H_4), 7.37, 7.43 (2 t_{br}, *J* ≈ 7.3 Hz, 1 H, 2 H, Ph), 7.48 (d_{br}, *J* ≈ 7.3 Hz, 2 H, Ph), 7.56 (d_{br}, *J* = 8.9 Hz, 2 H, C_6H_4), 7.70–7.76 (m, 2 H, C_6H_4), 8.07, 8.12 (2 d, *J* = 7.8 Hz, 1 H each, C_6H_4), 8.71, 8.82 (2 s, C_6H_2) ppm; ^{13}C NMR (151 MHz, $CDCl_3$): δ 70.4 (CH_2), 115.0 (2 CH), 119.2, 121.8 (2 *i*-C), 127.1, 127.6 (2 CH each), 128.3 (CH), 128.8 (2 CH), 129.6, 129.8 (2 *i*-C), 129.9, 130.1, 130.2, 130.4, 130.4, 130.4 (CH each), 134.8, 135.3, 136.3 (3 *i*-C), 140.8 (q, $^2J_{C,F}$ = 40.6 Hz, C-3), 160.0 (*i*-C), 174.4, 177.2 (2 C=O) ppm; ^{19}F NMR (565 MHz, $CDCl_3$): δ –62.73 ppm; UV (CH_2Cl_2), λ_{max} (log ε) 245 (4.86), 277 (4.48), 288 (4.56), 298 (4.51), 327 (3.88), 346 (3.81), 408 (4.00), 433 (3.80); IR (neat): ν 3071, 1681 (C=O), 1614, 1506, 1454, 1286, 1237, 1197, 1133, 1070, 947, 835, 764 cm^{-1}; ESI-MS (*m*/*z*):

499.5 (25, $[M + H]^+$), 521.4 (100 $[M + Na]^+$); elemental analysis calcd (%) for $C_{29}H_{17}F_3N_2O_3$ (498.5): C 69.88, H 3.44, N 5.62; found: C 69.66, H 3.51, N 5.75.

1-(4-Methoxyphenyl)-3-(trifluoromethyl)-1*H*-naphtho[2,3-*f*]indazole-4,11-dione (**9j**)

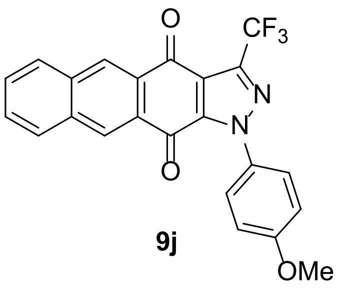

9j

Reaction time: 2 d, 313 mg (74%), yellow solid, mp 295–296 °C. ^{1}H NMR (600 MHz, $CDCl_3$): δ 3.92 (s, 3 H, OMe), 7.07, 7.56 (2 d_{br}, J = 8.9 Hz, 2 H each, C_6H_4), 7.70–7.76 (m, 2 H), 8.07, 8.12 (2 d_{br}, $J \approx 7.8$ Hz, 1 H each, C_6H_4), 8.71, 8.82 (2 s, 1 H each, C_6H_2) ppm; ^{13}C NMR (151 MHz, $CDCl_3$): δ 55.7 (OMe), 114.1 (2 CH), 121.8 (*i*-C), 127.1 (2 CH), 129.6, 129.8 (2 *i*-C), 129.9, 130.1, 130.2, 130.3, 130.4, 130.4 (CH each), 131.2, 134.8, 135.3, 139.8 (4 *i*-C), 140.7 (q, $^2J_{C,F}$ = 40.7 Hz, C-3), 160.8 (*i*-C), 174.3, 177.2 (2 C=O) ppm; ^{19}F NMR (565 MHz, $CDCl_3$): δ –62.7 ppm; UV (CH_2Cl_2), λ_{max} (log ε) 243 (4.78), 277 (4.47), 287 (4.56), 299 (4.47), 324 (3.85), 345 (3.76), 407 (3.99), 431 (3.80); IR (neat): ν 3019, 1681 (C=O), 1618, 1506, 1446, 1290, 1252, 1197, 1122, 1025, 947, 835, 760 cm^{-1}; ESI-MS (*m*/*z*): 423.3 (10, $[M + H]^+$), 445.3 (39, $[M + Na]^+$);

elemental analysis calcd (%) for $C_{23}H_{13}F_3N_2O_3$ (422.4): C 65.41, H 3.10, N 6.63; found: C 65.37, H 3.12, N 6.64.

1-(*p*-Tolyl)-3-(trifluoromethyl)-1*H*-naphtho[2,3-*f*]indazole-4,11-dione (**9k**)

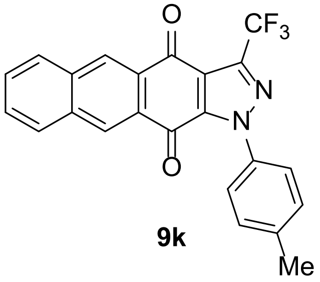

9k

Reaction time: 2 d, 374 mg (92%), yellow solid, mp 329–330 °C. ^{1}H NMR (600 MHz, $CDCl_3$): δ 2.50 (s, 3 H, Me), 7.38, 7.51 (2 d, $J \approx 8.2$ Hz, 2 H each, Tol), 7.70–7.76 (m, 2H), 8.07, 8.12 (2 d_{br}, $J \approx 7.8$ Hz, 1 H each, C_6H_4), 8.71, 8.82 (2 s, 1 H each, C_6H_2) ppm; ^{13}C NMR (151 MHz, $CDCl_3$): δ 21.4 (Me), 121.9 (*i*-C), 125.6 (2 CH), 129.6 (*i*-C), 129.6 (2 CH), 129.9, 130.1, 130.2, 130.3, 130.4, 130.4 (CH each), 134.8, 135.3, 135.8, 139.9, 140.6 (5 *i*-C), 174.3, 177.2 (2 C=O) ppm; ^{19}F NMR (565 MHz, $CDCl_3$): δ –62.7 ppm; UV (CH_2Cl_2), λ_{max} (log ε) 243 (4.68), 277 (4.41), 289 (4.50), 299 (4.42), 325 (3.91), 341 (3.71), 387 (3.76), 407 (3.88), 433 (3.63); IR (neat): ν 2926, 2851, 1681 (C=O), 1618, 1509, 1450, 1286, 1238, 1197, 1118, 1074, 947, 828, 760 cm^{-1}; ESI-MS (*m*/*z*): 429.3 (35, $[M + Na]^+$), 445.2 (100, $[M + K]^+$); elemental analysis calcd (%) for $C_{23}H_{13}F_3N_2O_2$ (406.4): C 67.98, H 3.22, N 6.89; found: C 68.07, H 3.24, N 6.85.

1-(4-Chlorophenyl)-3-(trifluoromethyl)-1*H*-naphtho[2,3-*f*]indazole-4,11-dione (**9l**)

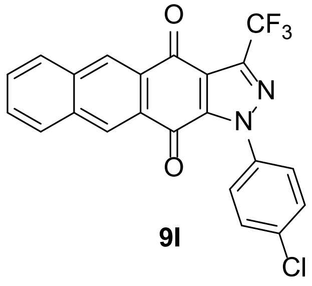

9l

Reaction time: 2 d, 269 mg (63%), yellow solid, mp 336–337 °C. ^{1}H NMR (600 MHz, $C_2D_2Cl_4$, 70 °C): δ 7.61, 7.66 (2 d, *J* ≈ 8.7 Hz, 2 H each, C_6H_4), 7.74–7.83 (m, 2H), 8.12, 8.16 (2 d_{br}, *J* ≈ 7.6 Hz, 1 H each, C_6H_4), 8.77, 8.86 (2s, 1 H each, C_6H_2) ppm; ^{13}C NMR (151 MHz, $C_2D_2Cl_4$, 70 °C): δ 99.3 (*i*-C), 126.8, 128.9 (2 CH each), 129.2, 129.5 (2 *i*-C), 129.7, 129.7, 129.9, 130.0, 130.0, 130.1 (CH each), 134.5, 135.0, 136.0, 136.5, 139.9 (5 *i*-C), 173.8, 176.4 (2 C=O) ppm; ^{19}F NMR (565 MHz, $CDCl_3$): δ –62.8 ppm; UV (CH_2Cl_2), λ_{max} (log ε) 244 (4.62), 279 (4.38), 291 (4.66), 299 (4.39), 335 (3.69), 388 (3.63), 411 (3.75), 435 (3.54); IR (neat): ν 3068, 1685 (C=O), 1614, 1528, 1498, 1454, 1405, 1290, 1200, 1126, 1074, 947, 835, 760 cm^{-1}; ESI-MS (*m*/*z*): 449.2 (50, $[M + Na]^+$); elemental analysis calcd (%) for $C_{22}H_{10}ClF_3N_2O_2$ (426.8): C 61.92, H 2.36, N 6.56; found: C 61.67, H 2.36, N 6.68.

Copies of ^{1}H and ^{13}C NMR spectra

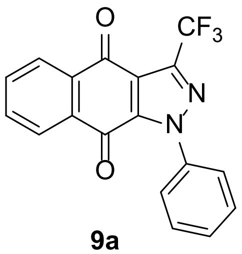

9a

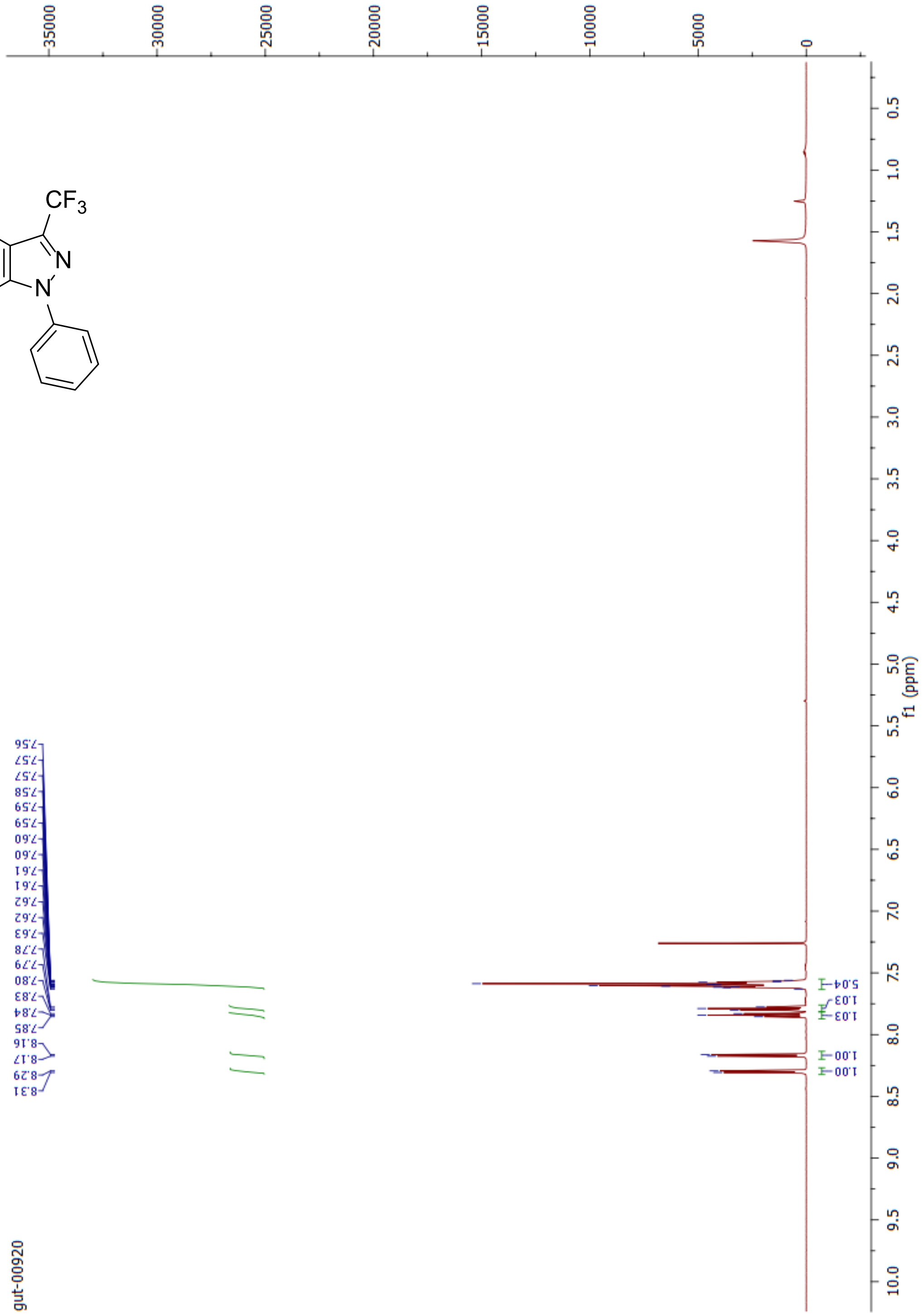

Figure S1. The ^{1}H NMR (600 MHz, $CDCl_3$) spectrum for compound **9a**.

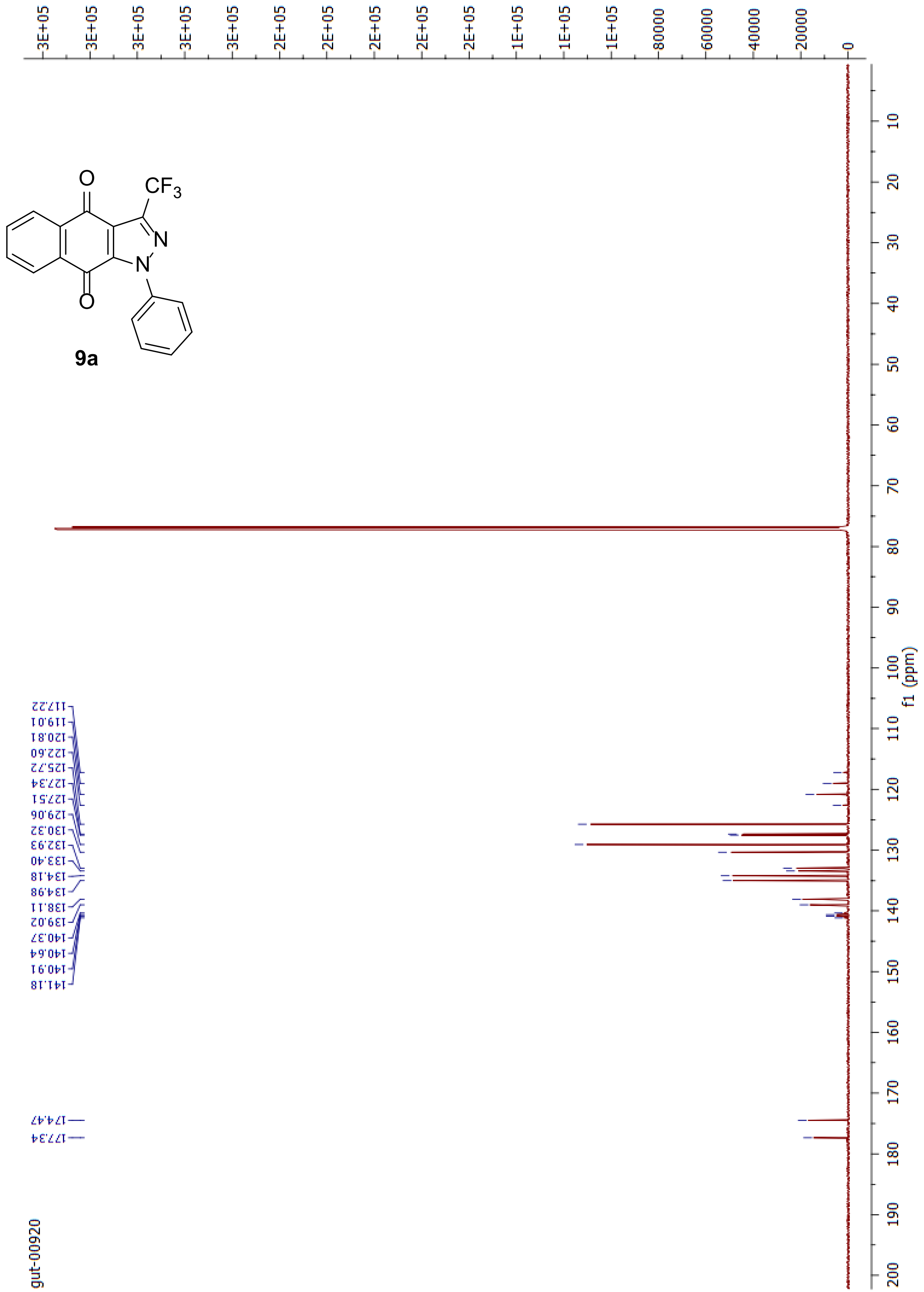

Figure S2. The ^{13}C NMR (151 MHz, $CDCl_3$) spectrum for compound **9a**.

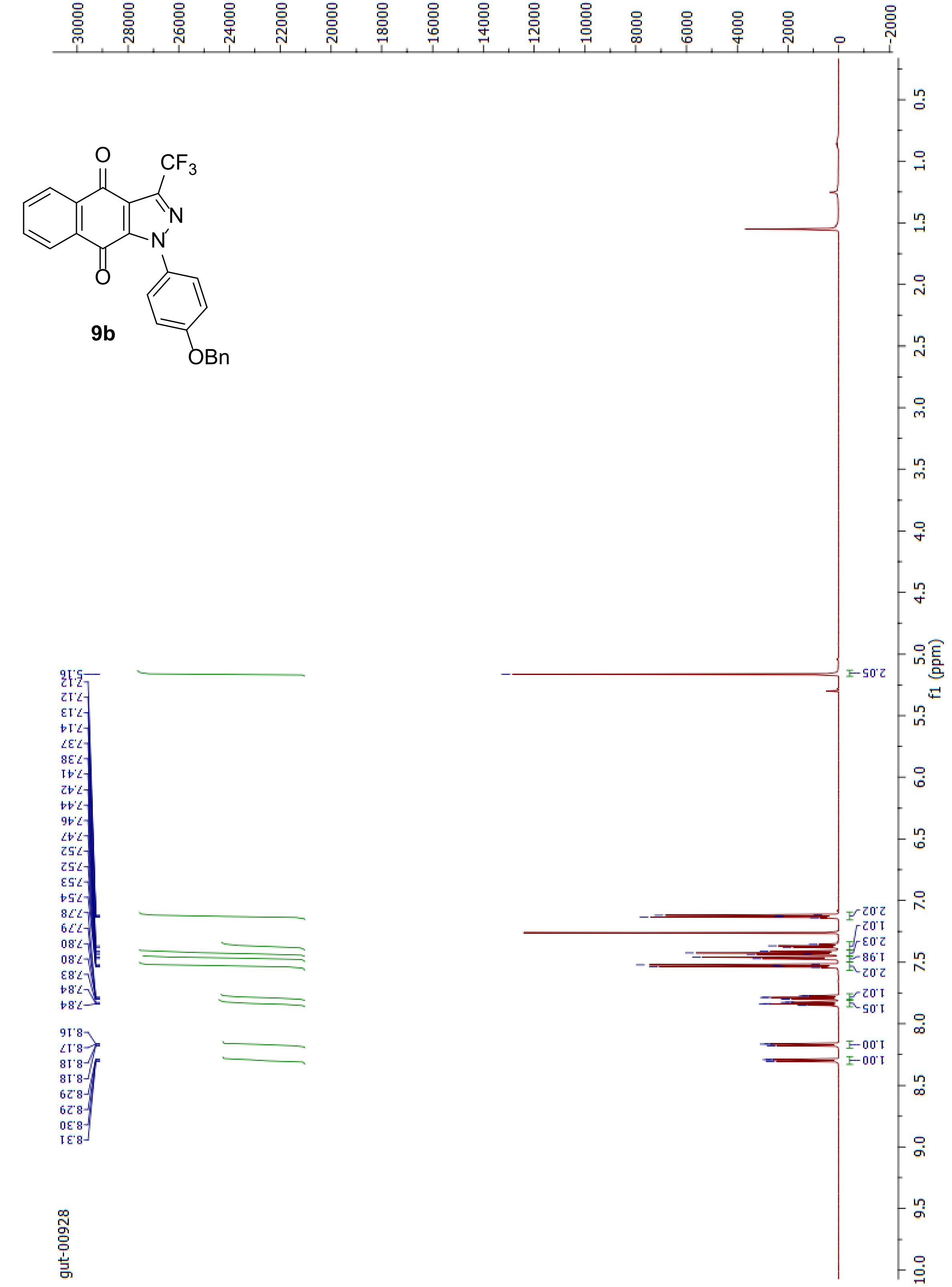

Figure S3. The ^{1}H NMR (600 MHz, $CDCl_3$) spectrum for compound **9b**.

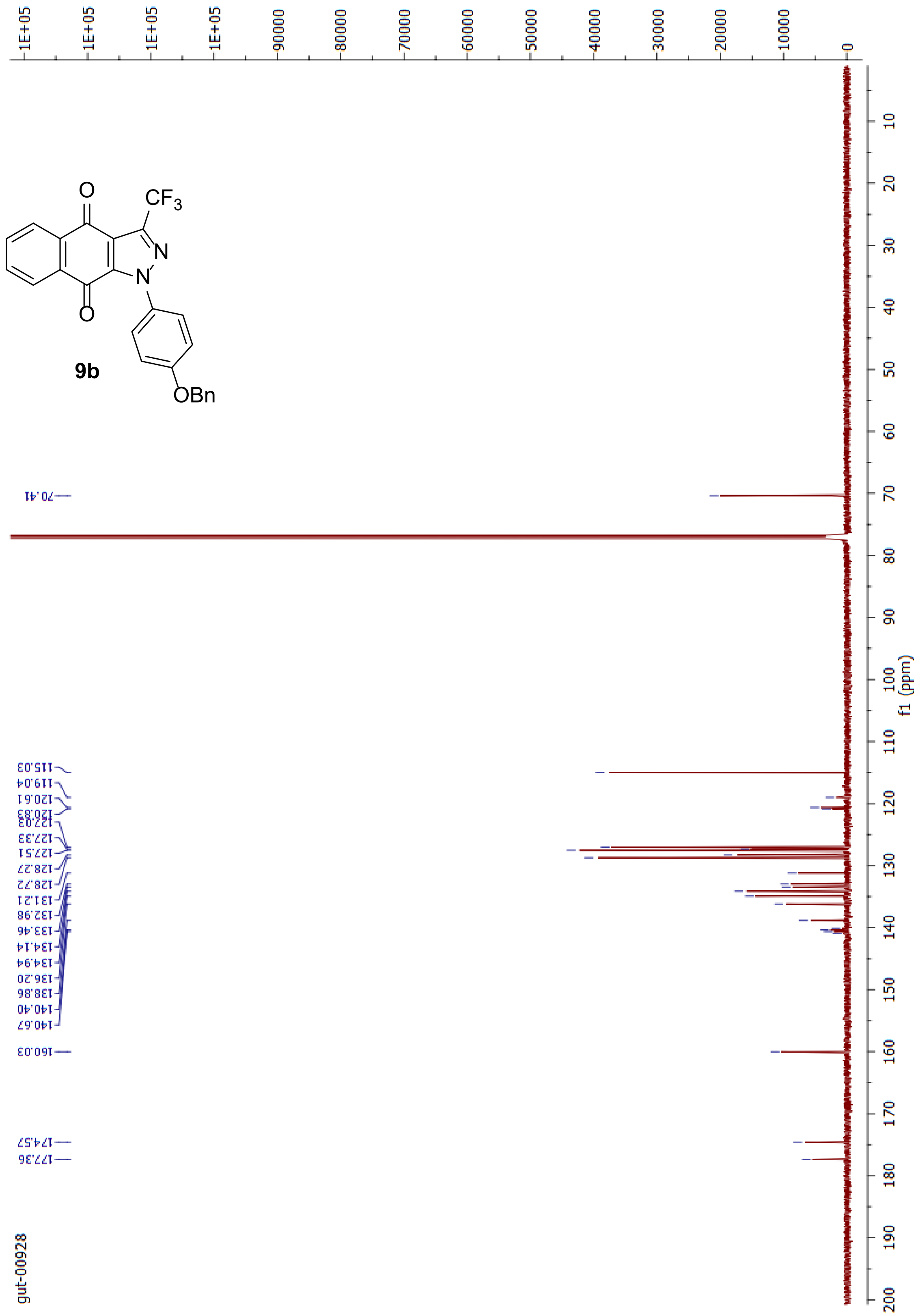

Figure S4. The ^{13}C NMR (151 MHz, $CDCl_3$) spectrum for compound **9b**.

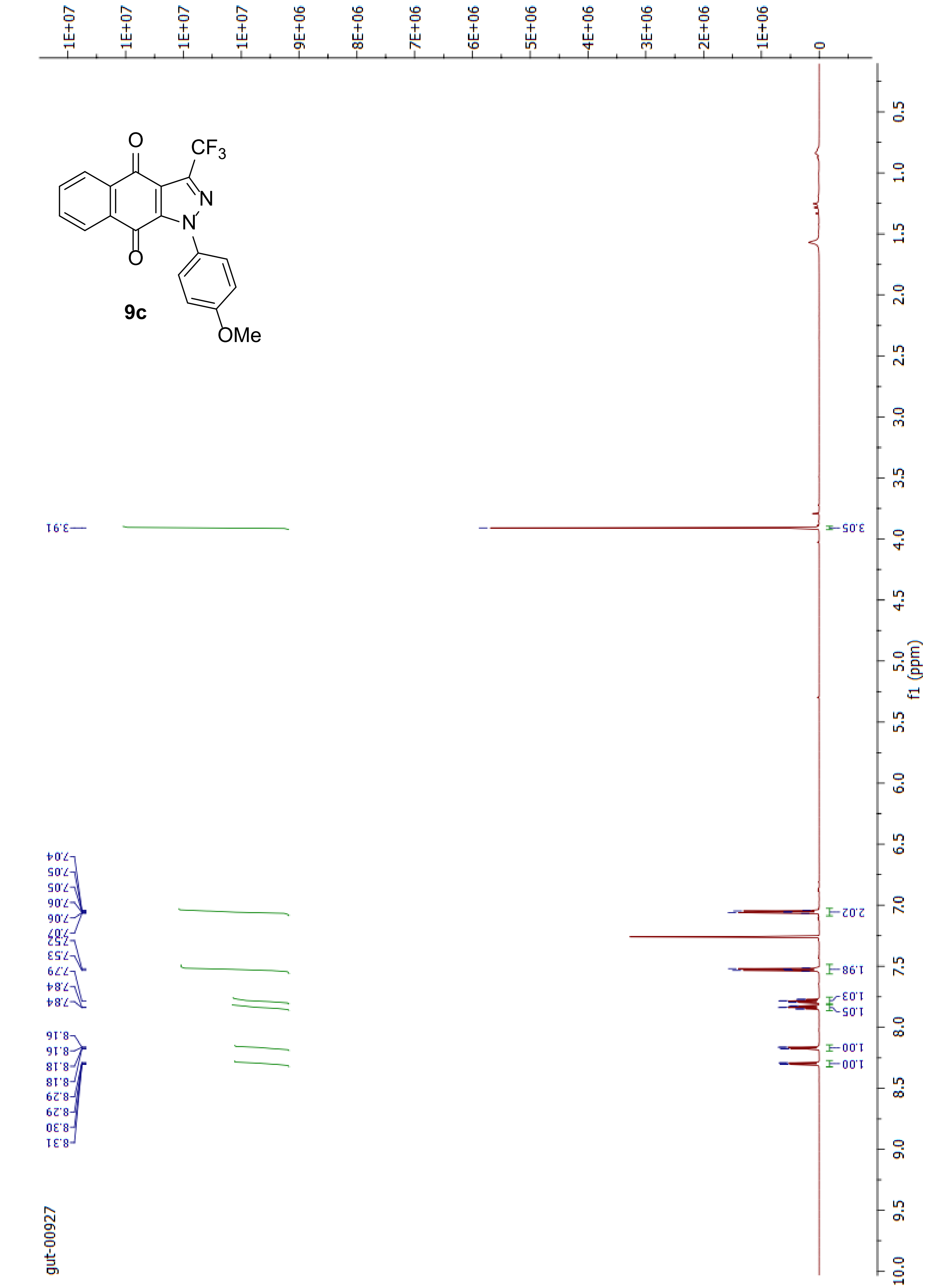

Figure S5. The ^{1}H NMR (600 MHz, $CDCl_3$) spectrum for compound **9c**.

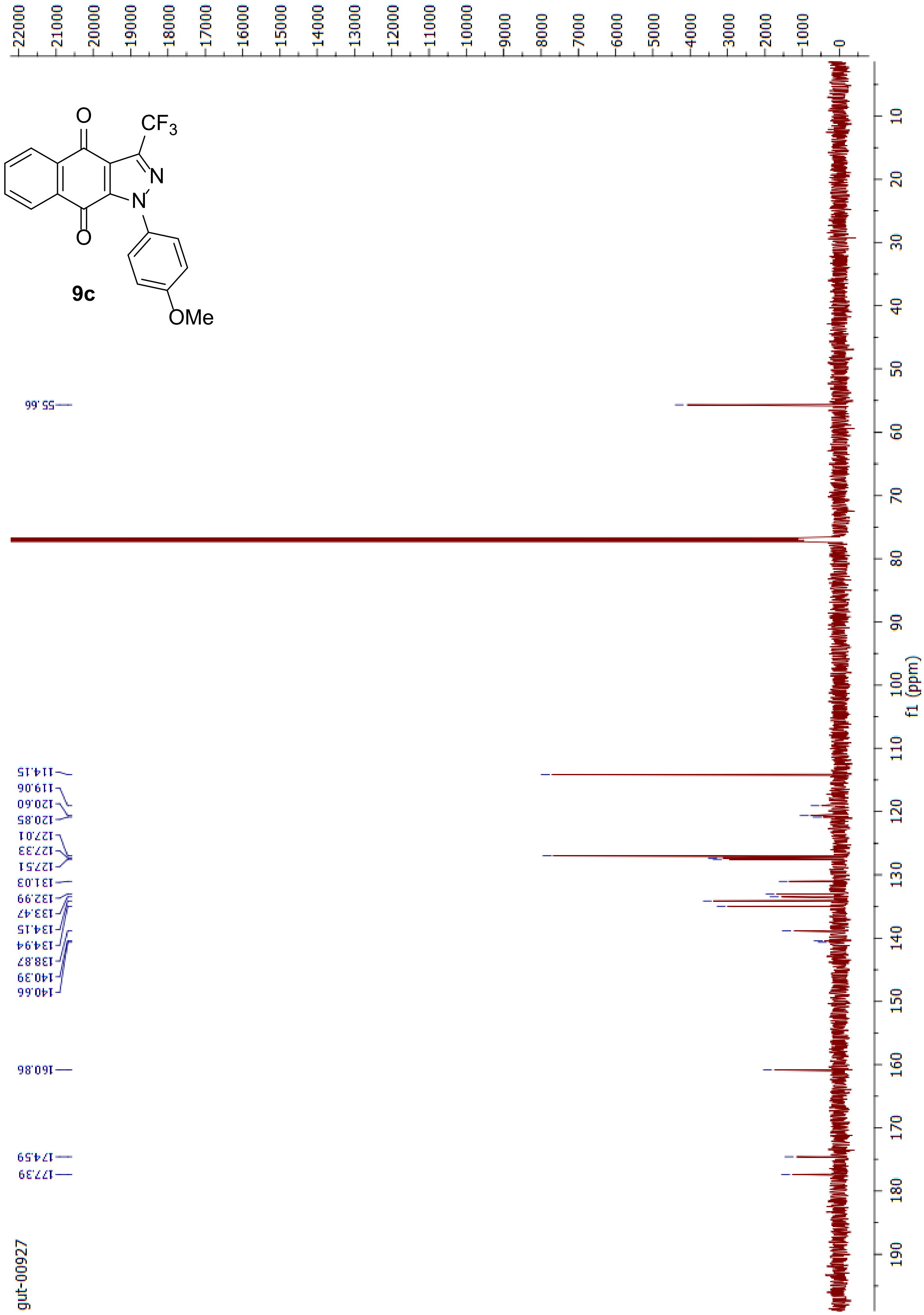

Figure S6. The ^{13}C NMR (151 MHz, $CDCl_3$) spectrum for compound **9c**.

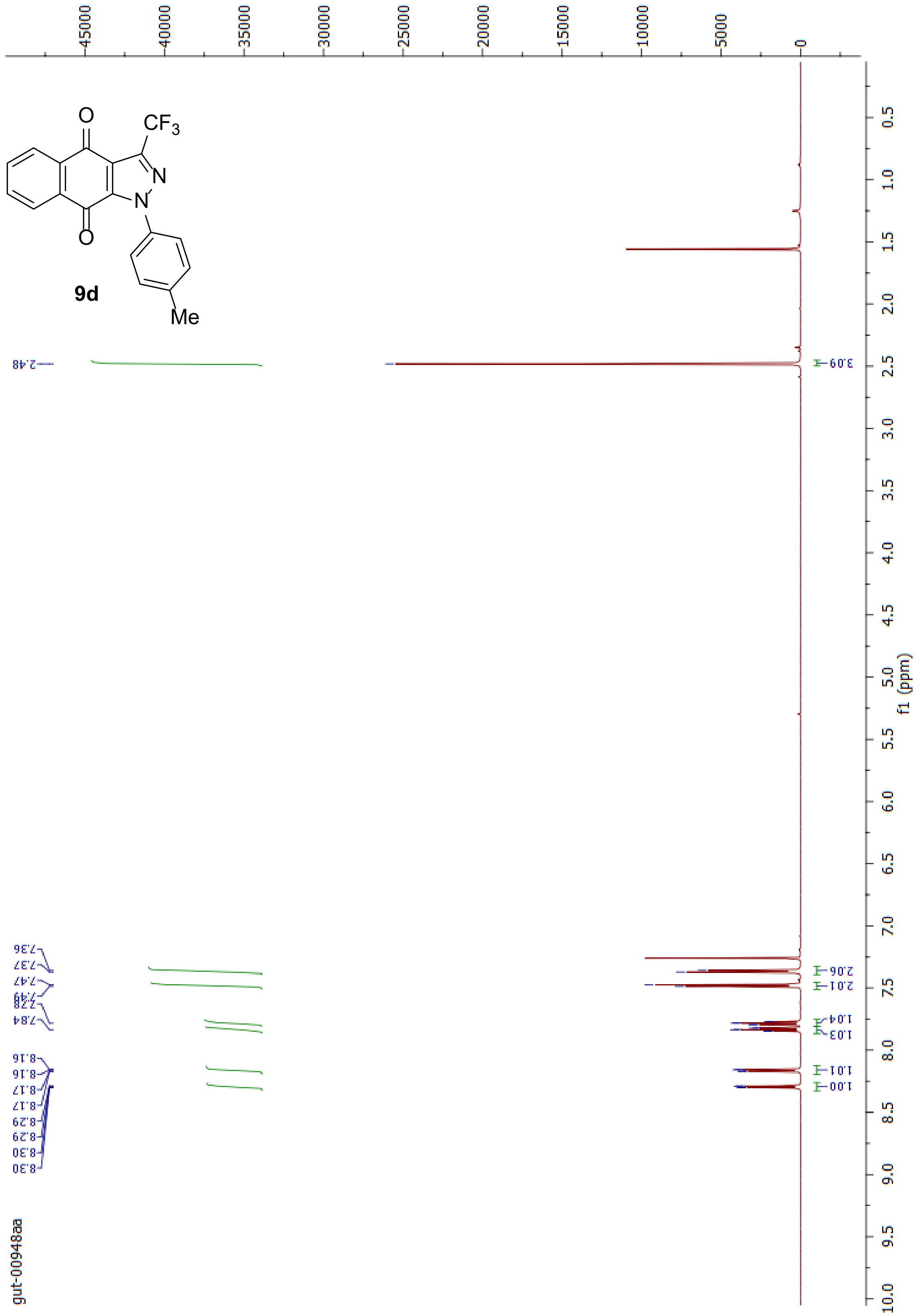

Figure S7. The ^{1}H NMR (600 MHz, $CDCl_3$) spectrum for compound **9d**.

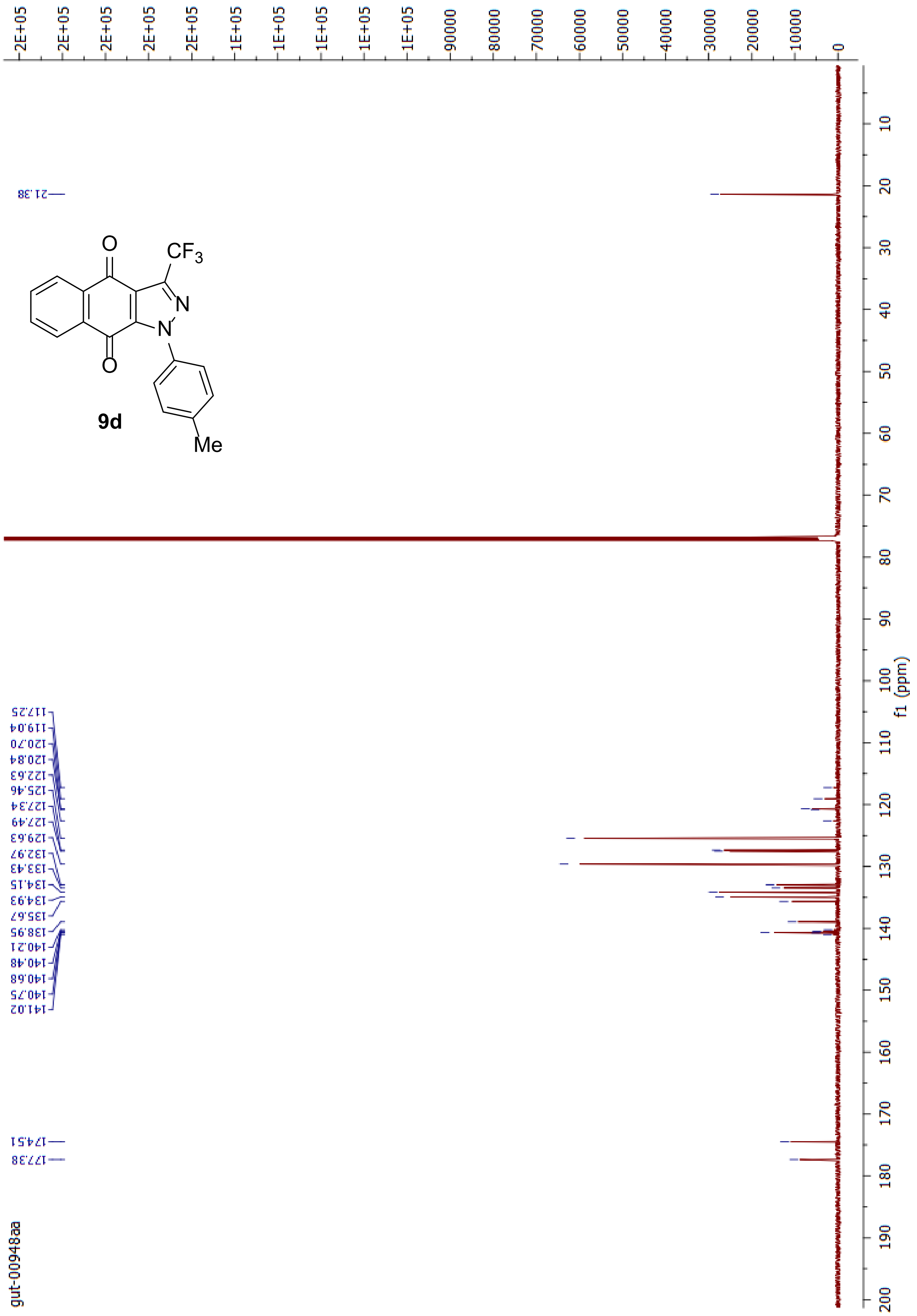

Figure S8. The ^{13}C NMR (151 MHz, $CDCl_3$) spectrum for compound **9d**.

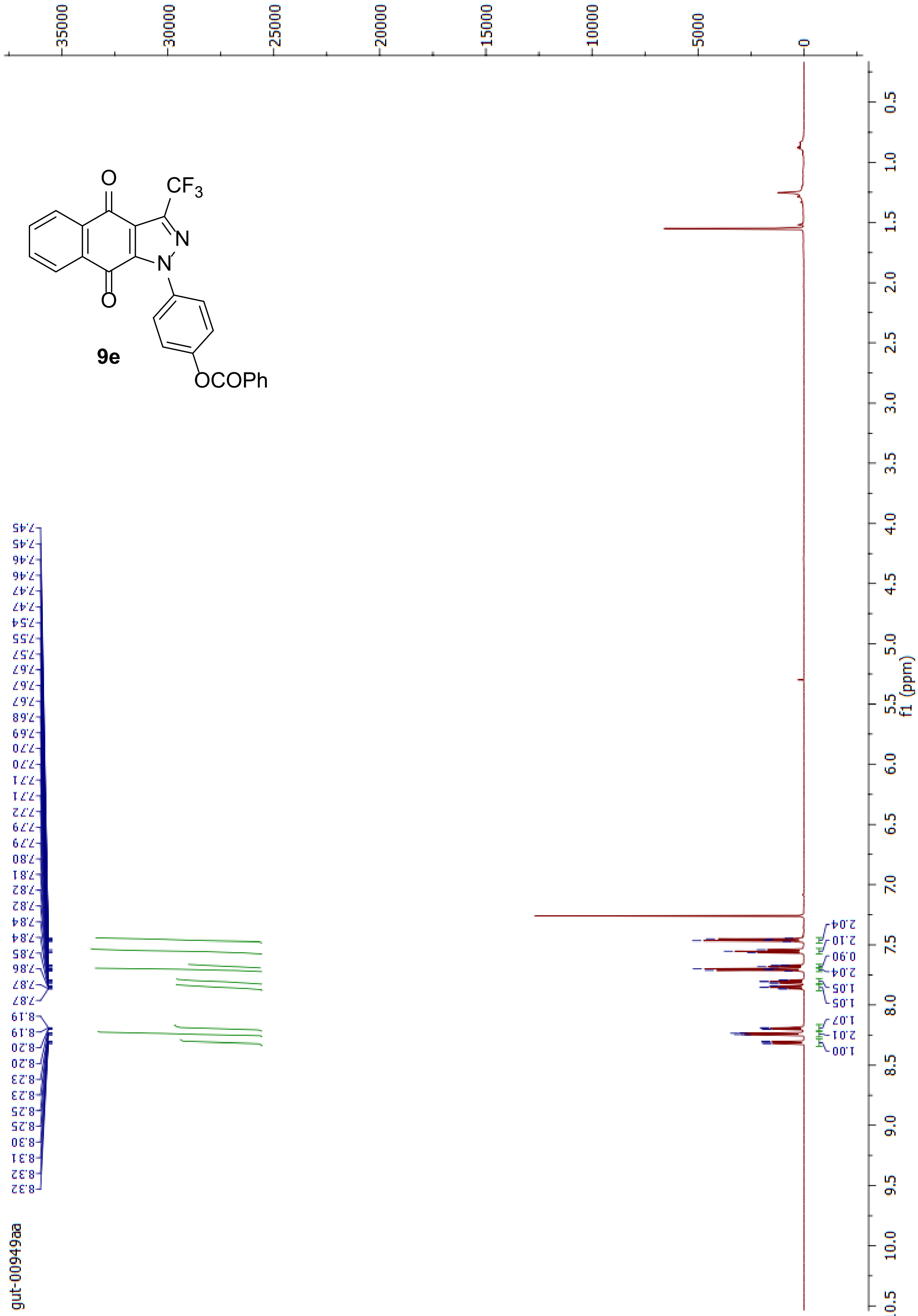

Figure S9. The ^{1}H NMR (600 MHz, $CDCl_3$) spectrum for compound **9e**.

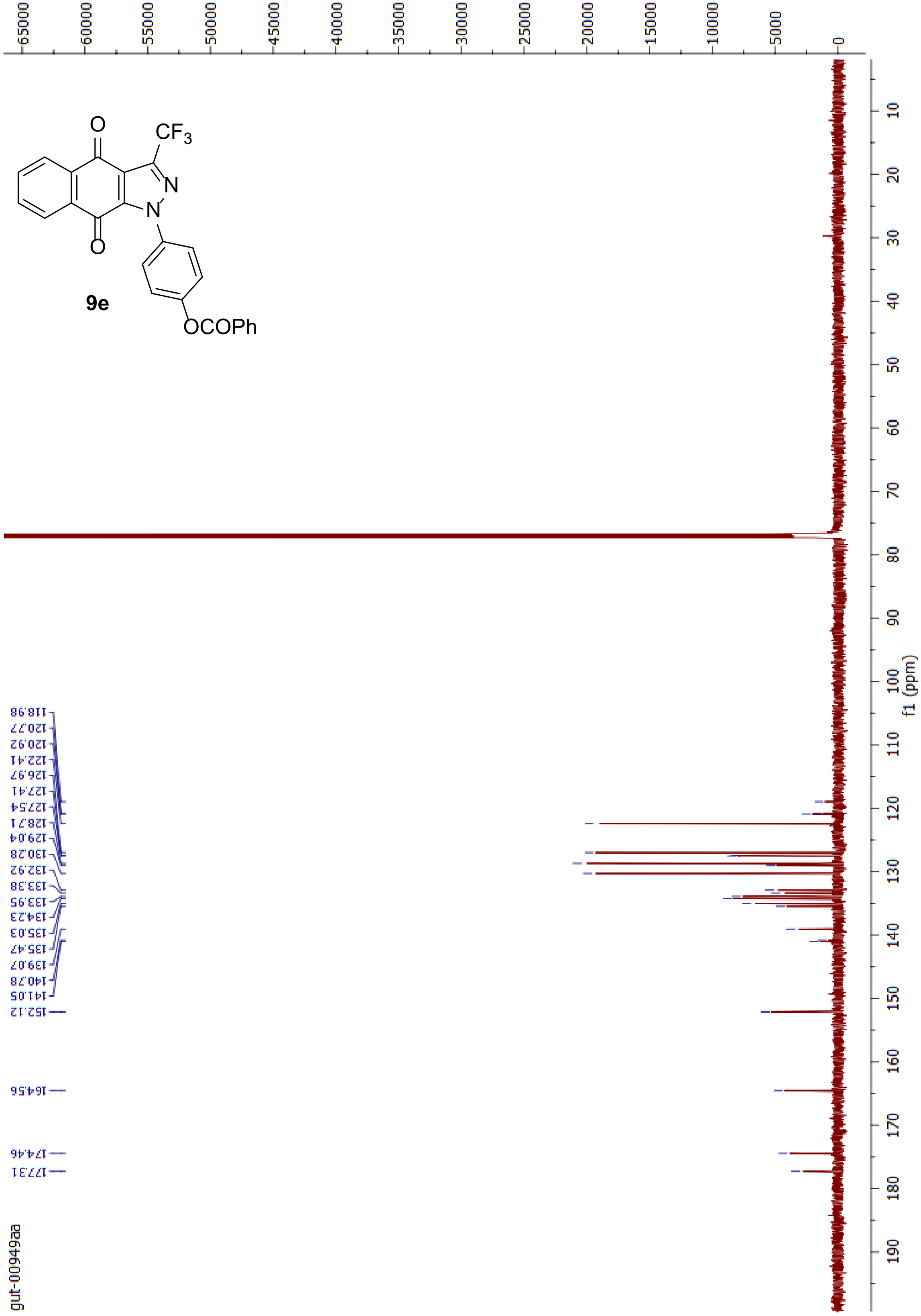

Figure S10. The ^{13}C NMR (151 MHz, $CDCl_3$) spectrum for compound **9e**.

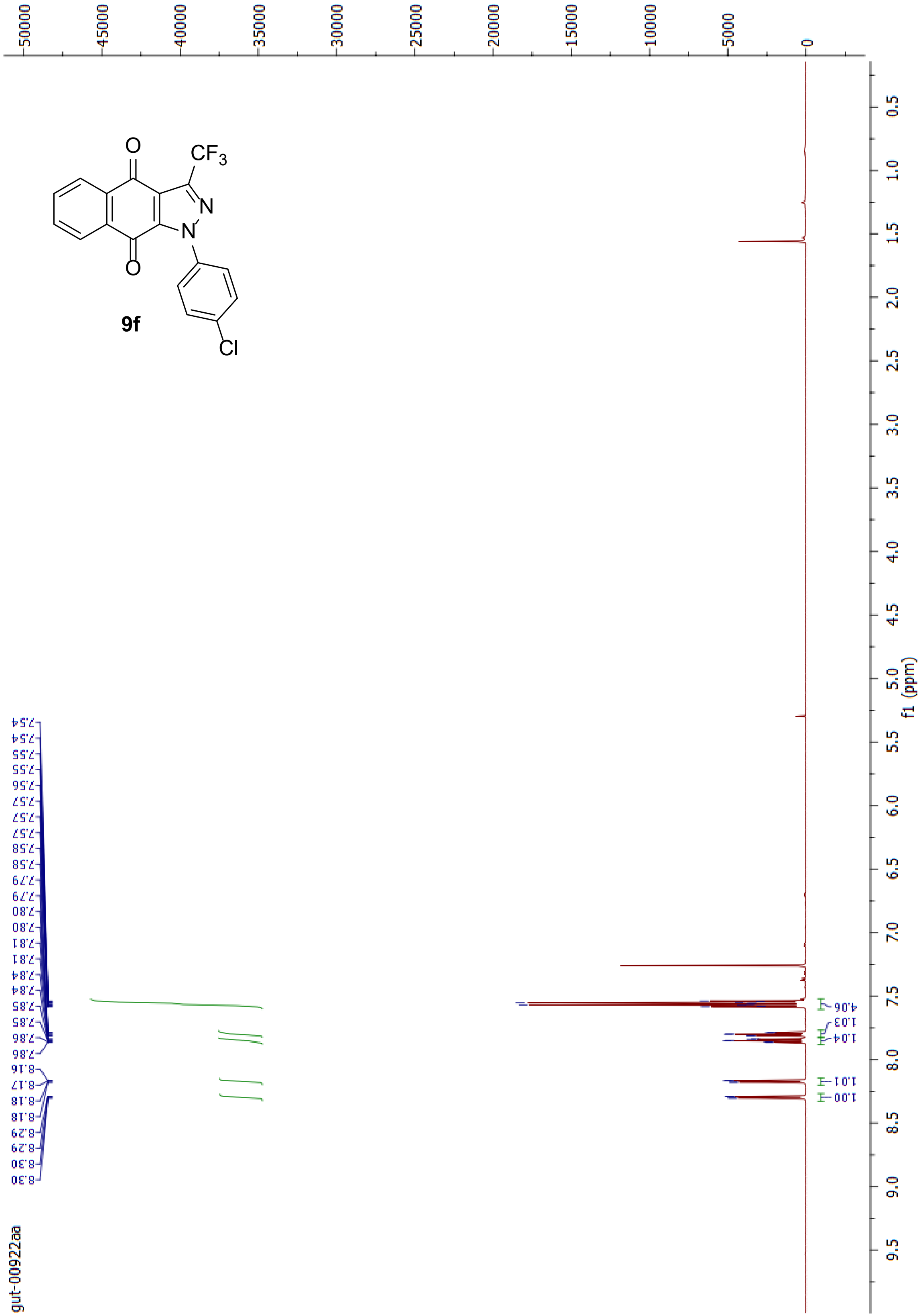

Figure S11. The ^1H NMR (600 MHz, $CDCl_3$) spectrum for compound **9f**.

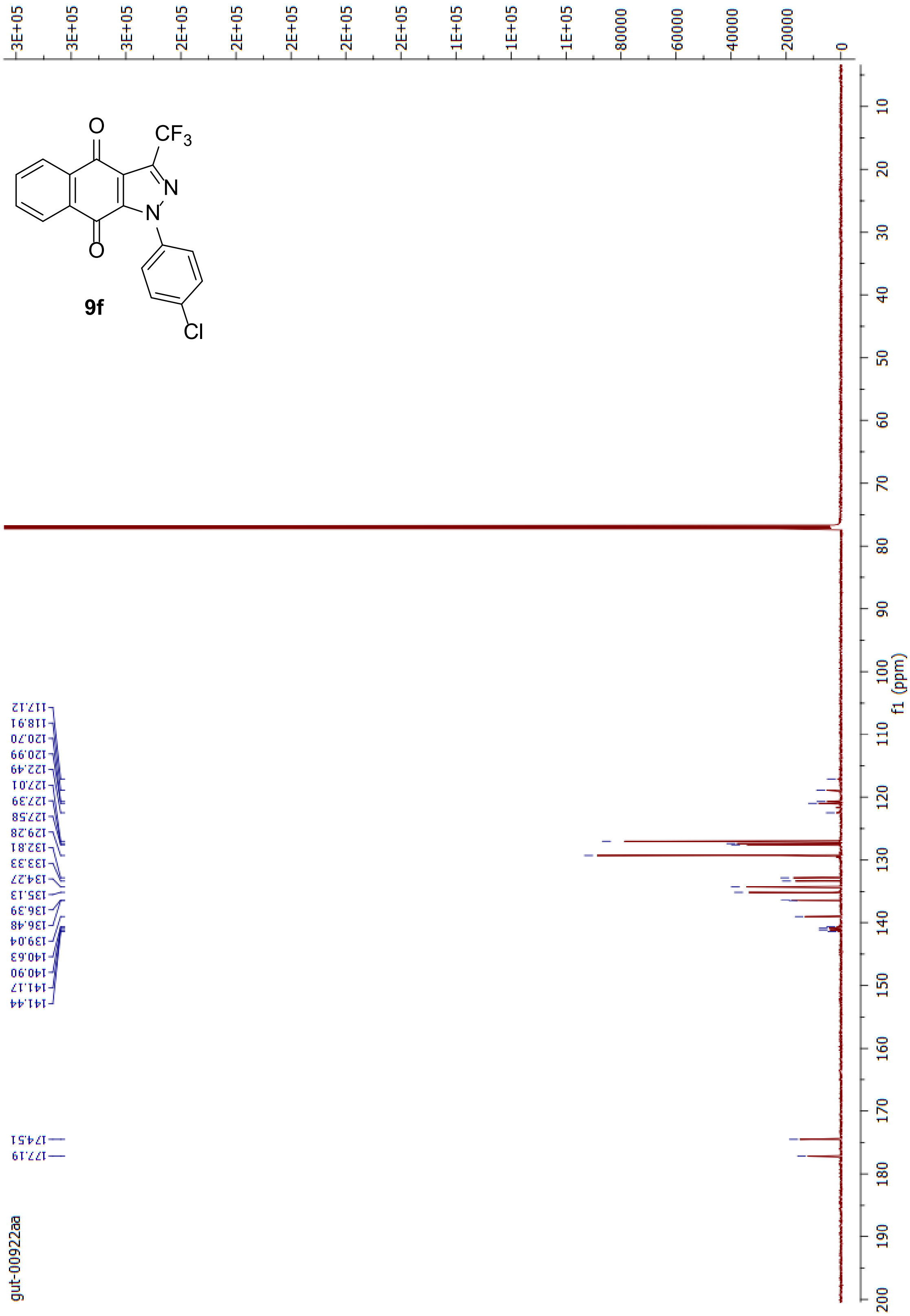

Figure S12. The ^{13}C NMR (151 MHz, $CDCl_3$) spectrum for compound **9f**.

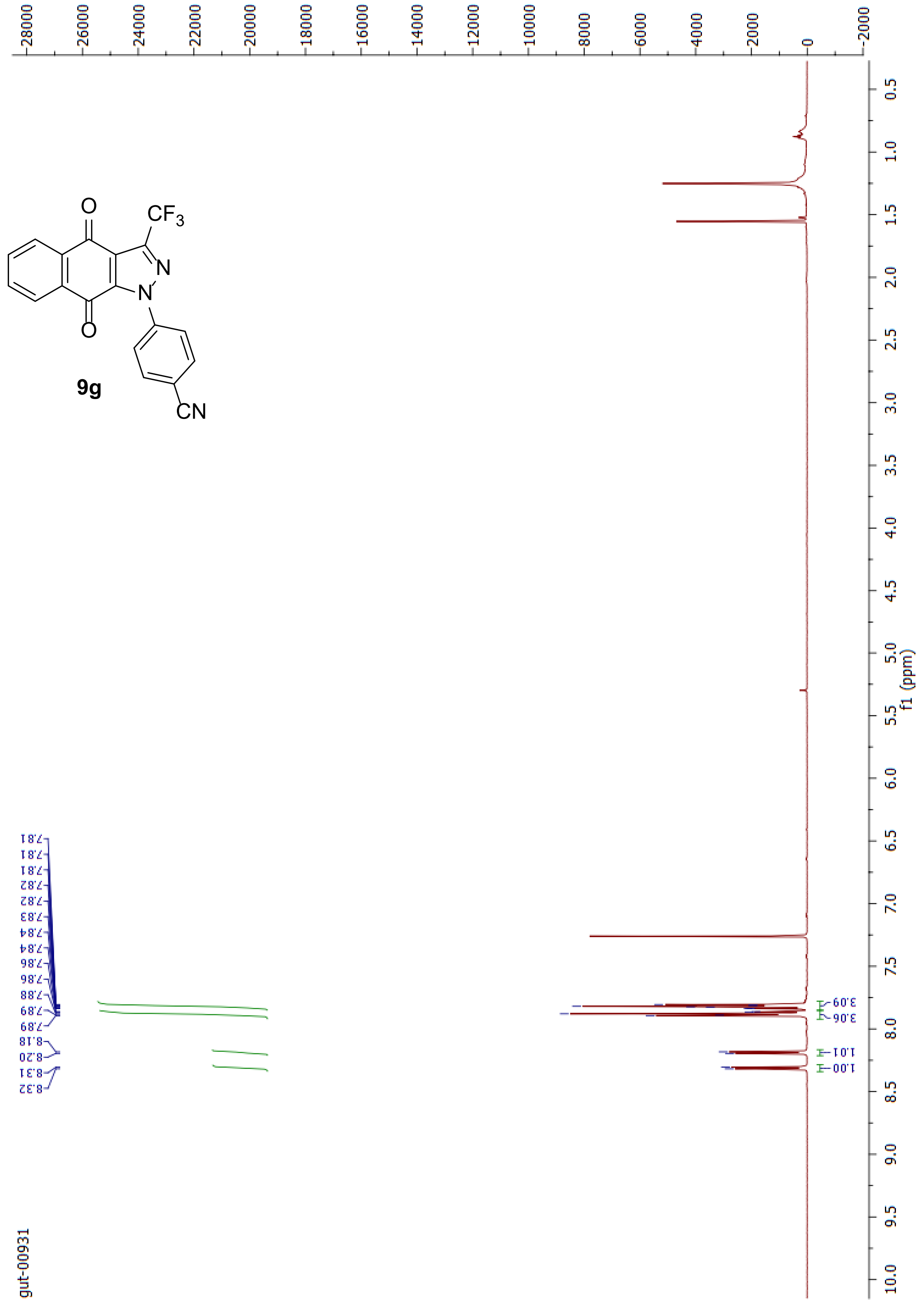

Figure S13. The ^{1}H NMR (600 MHz, $CDCl_3$) spectrum for compound **9g**.

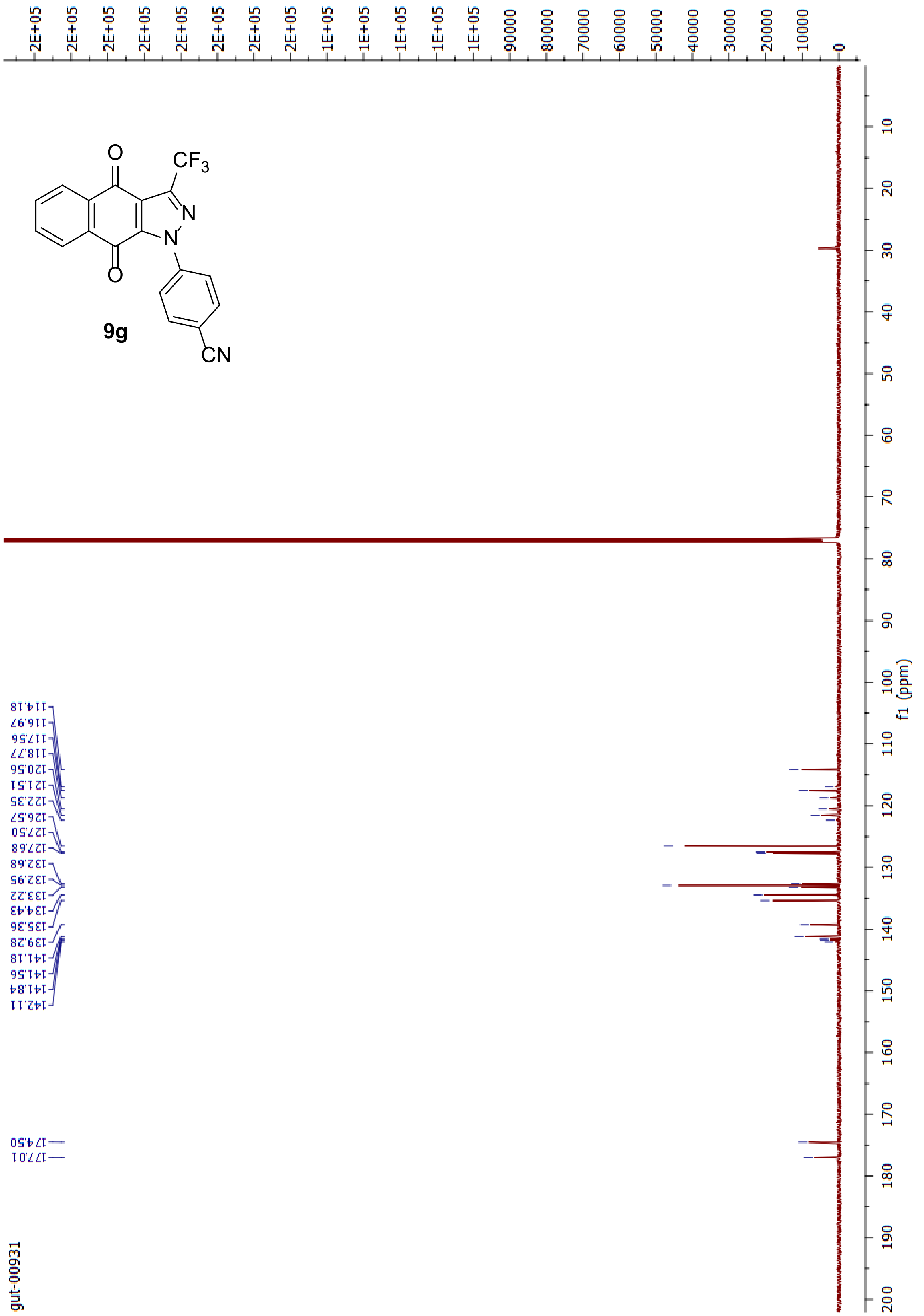

Figure S14. The ^{13}C NMR (151 MHz, $CDCl_3$) spectrum for compound **9g**.

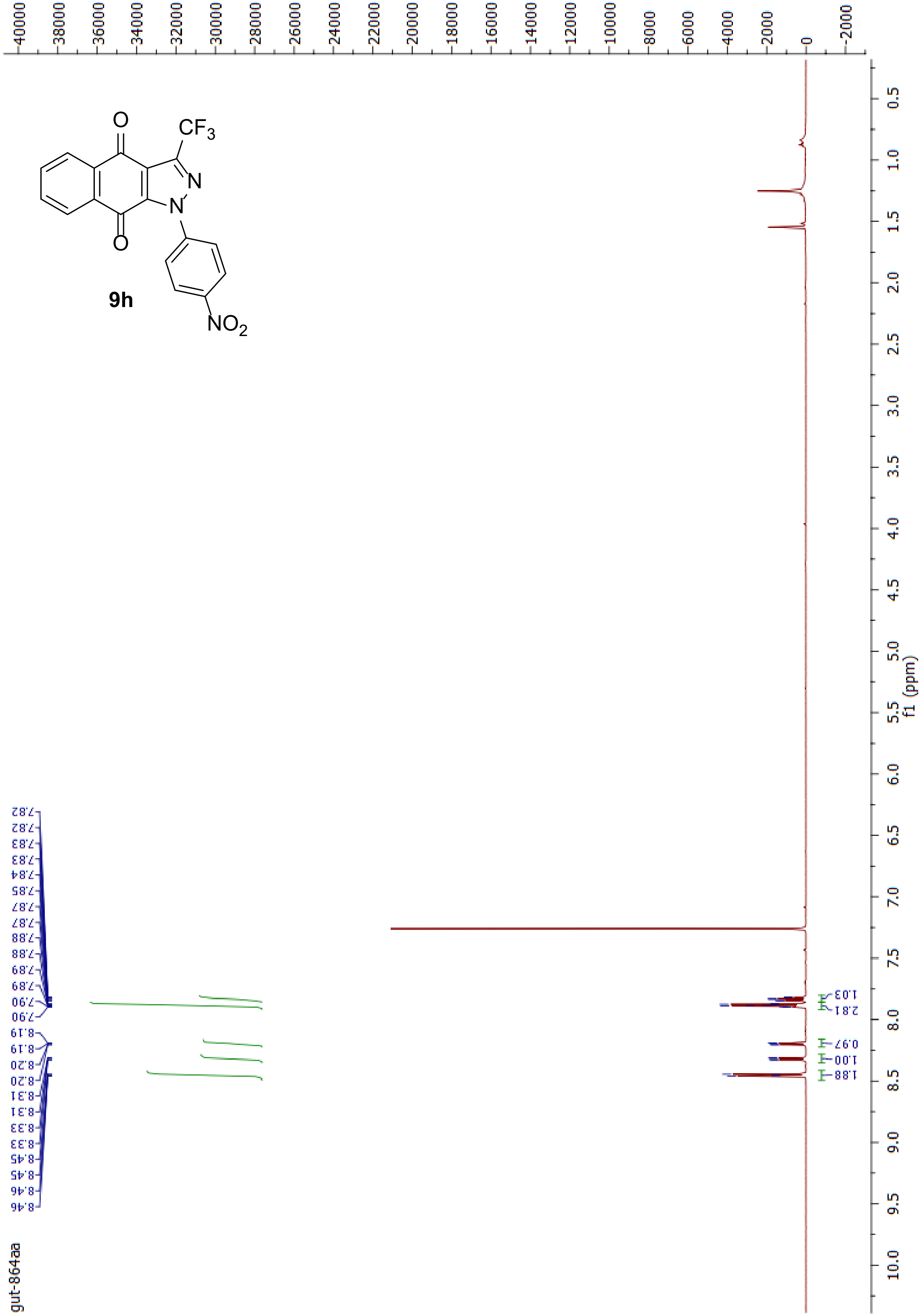

Figure S15. The ^{1}H NMR (600 MHz, $CDCl_3$) spectrum for compound **9h**.

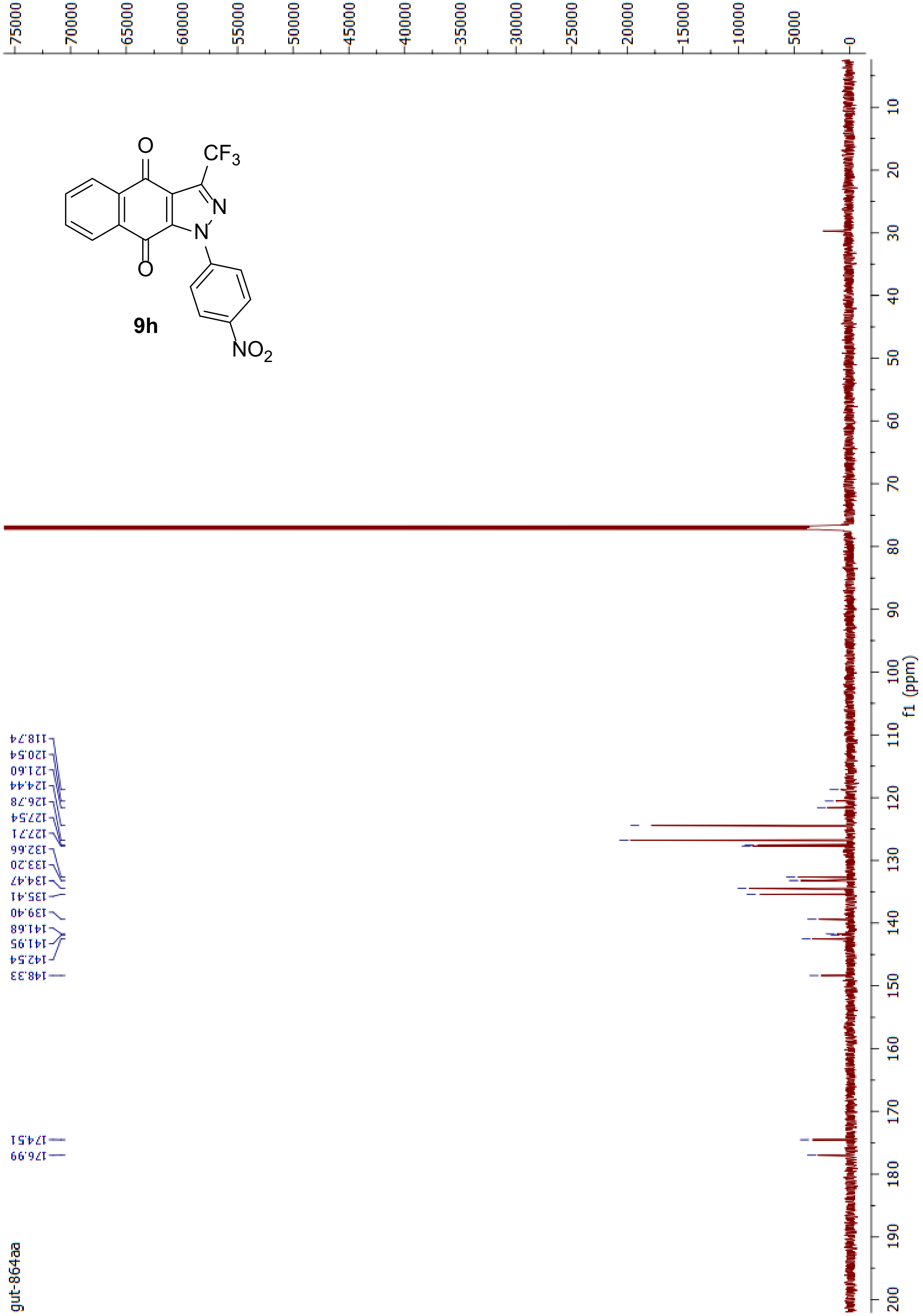

Figure S16. The ^{13}C NMR (151 MHz, $CDCl_3$) spectrum for compound **9h**.

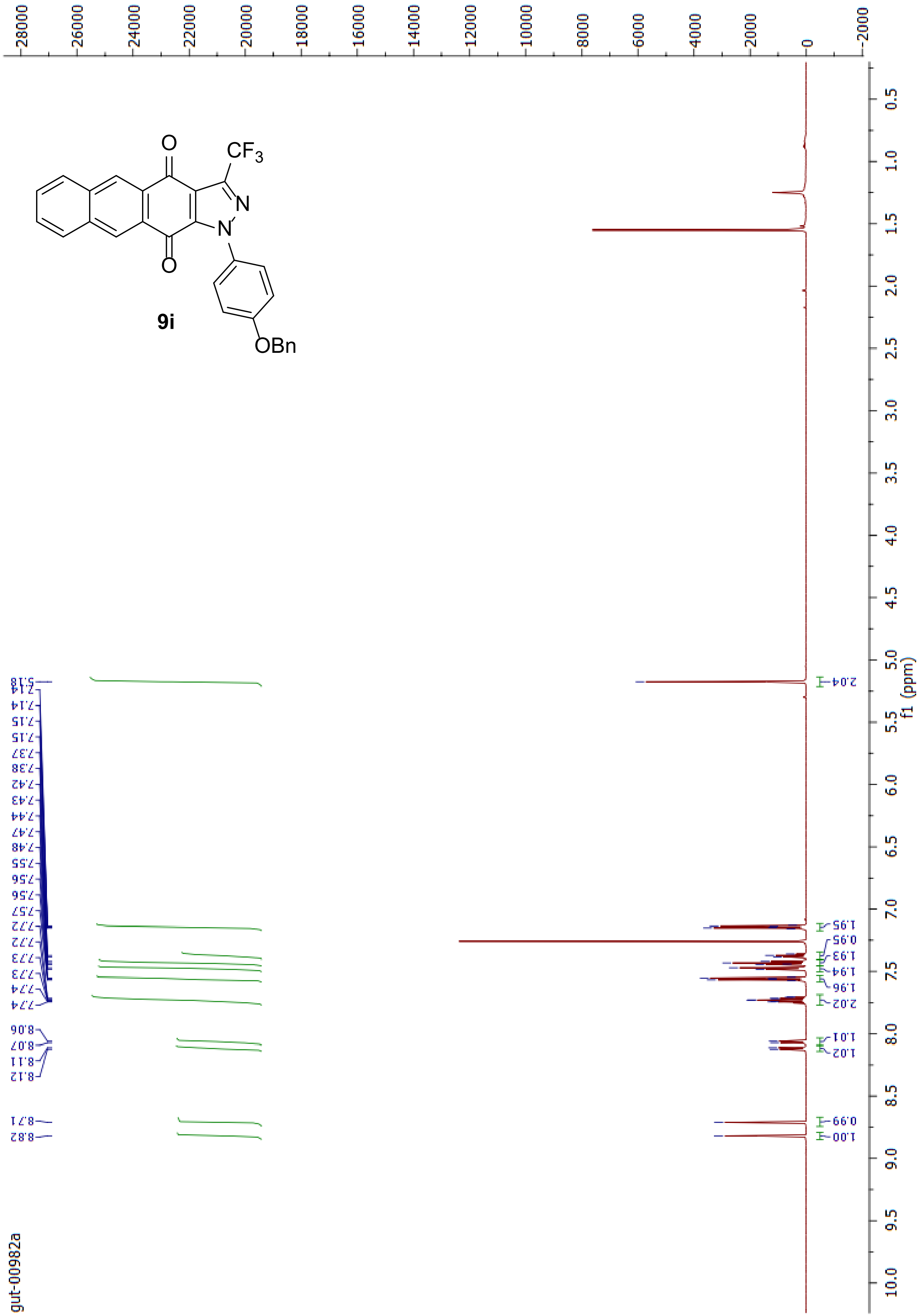

Figure S17. The ^{1}H NMR (600 MHz, $CDCl_3$) spectrum for compound **9i**.

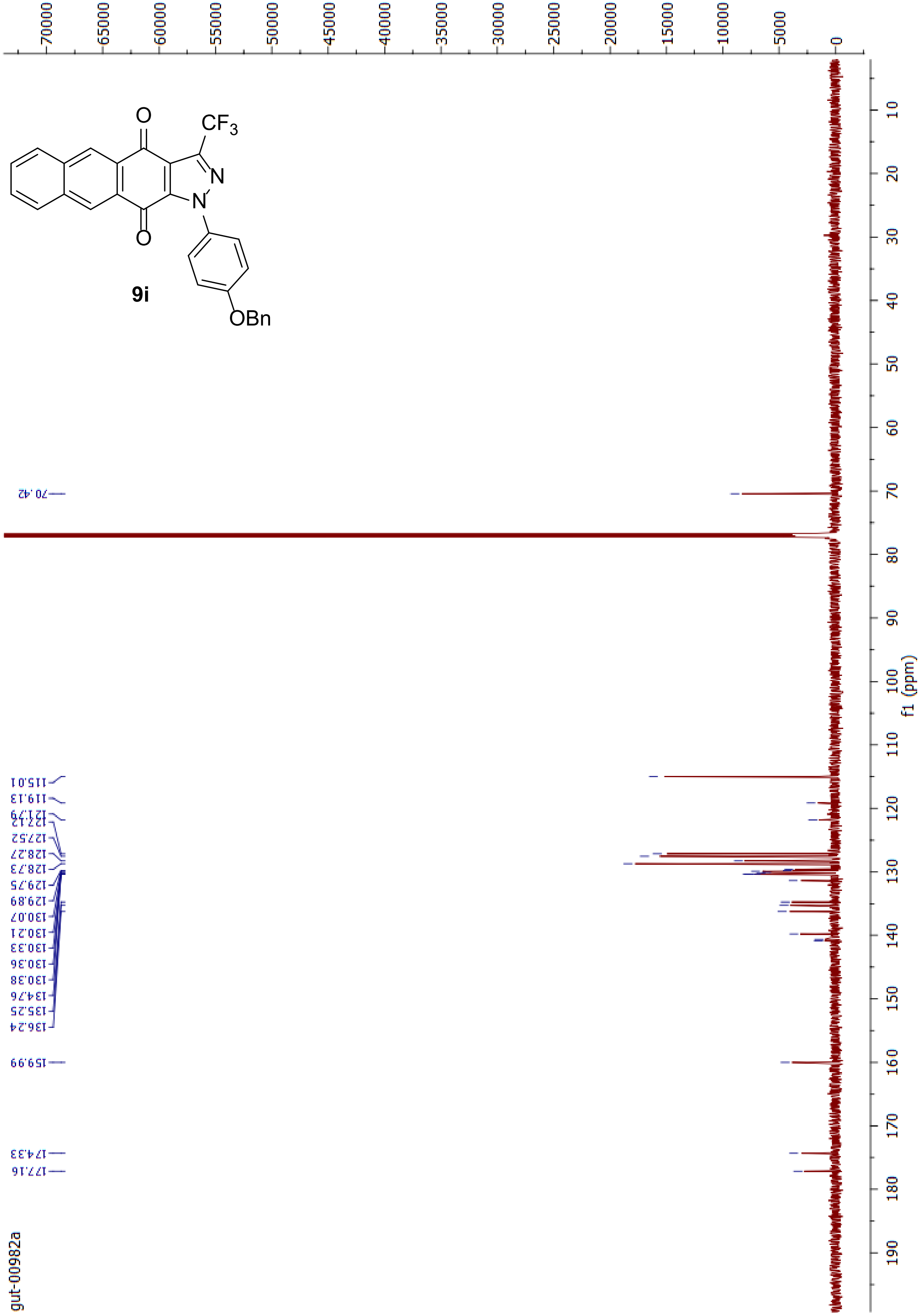

Figure S18. The ^{13}C NMR (151 MHz, $CDCl_3$) spectrum for compound **9i**.

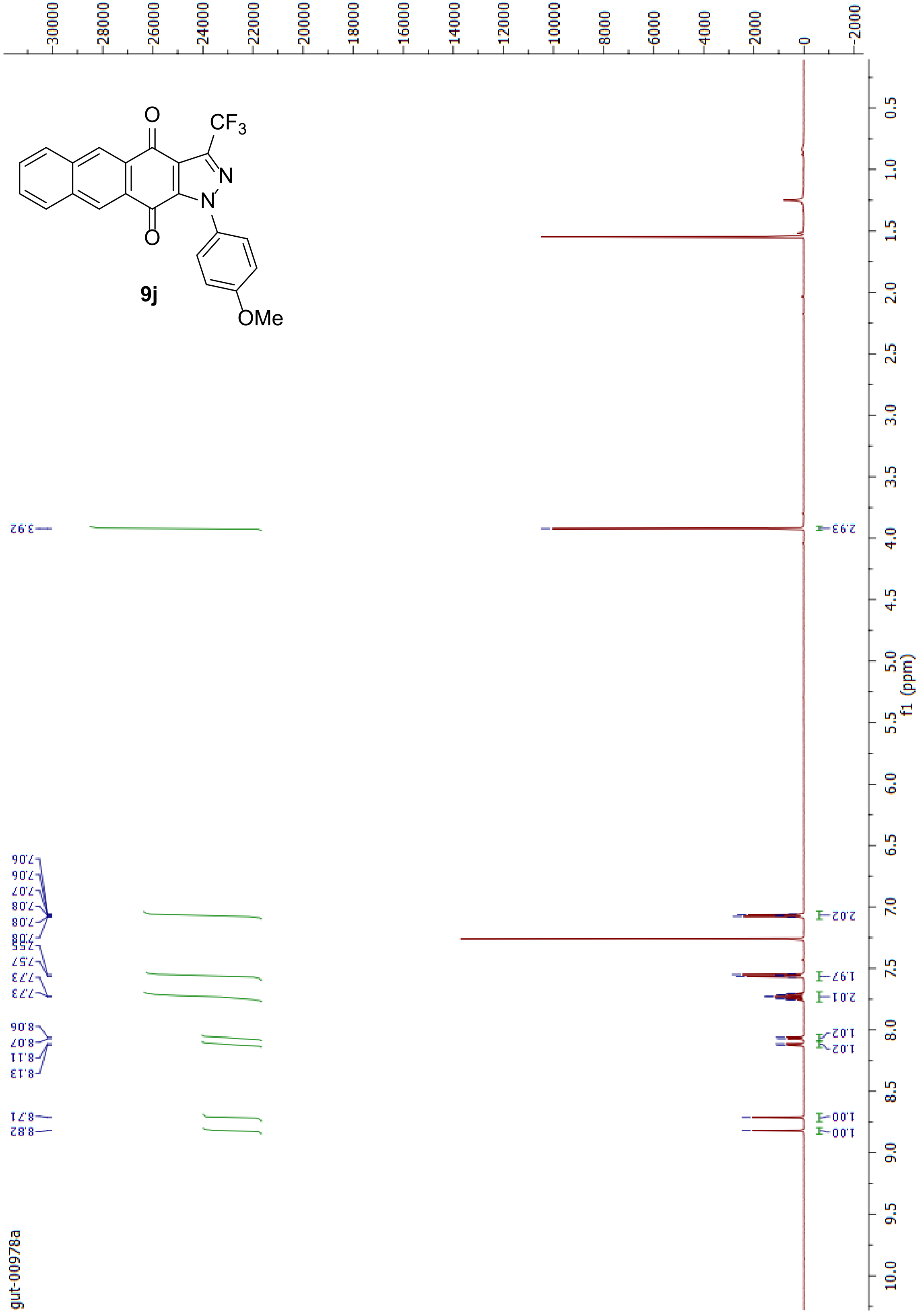

Figure S19. The ^{1}H NMR (600 MHz, $CDCl_3$) spectrum for compound **9j.**

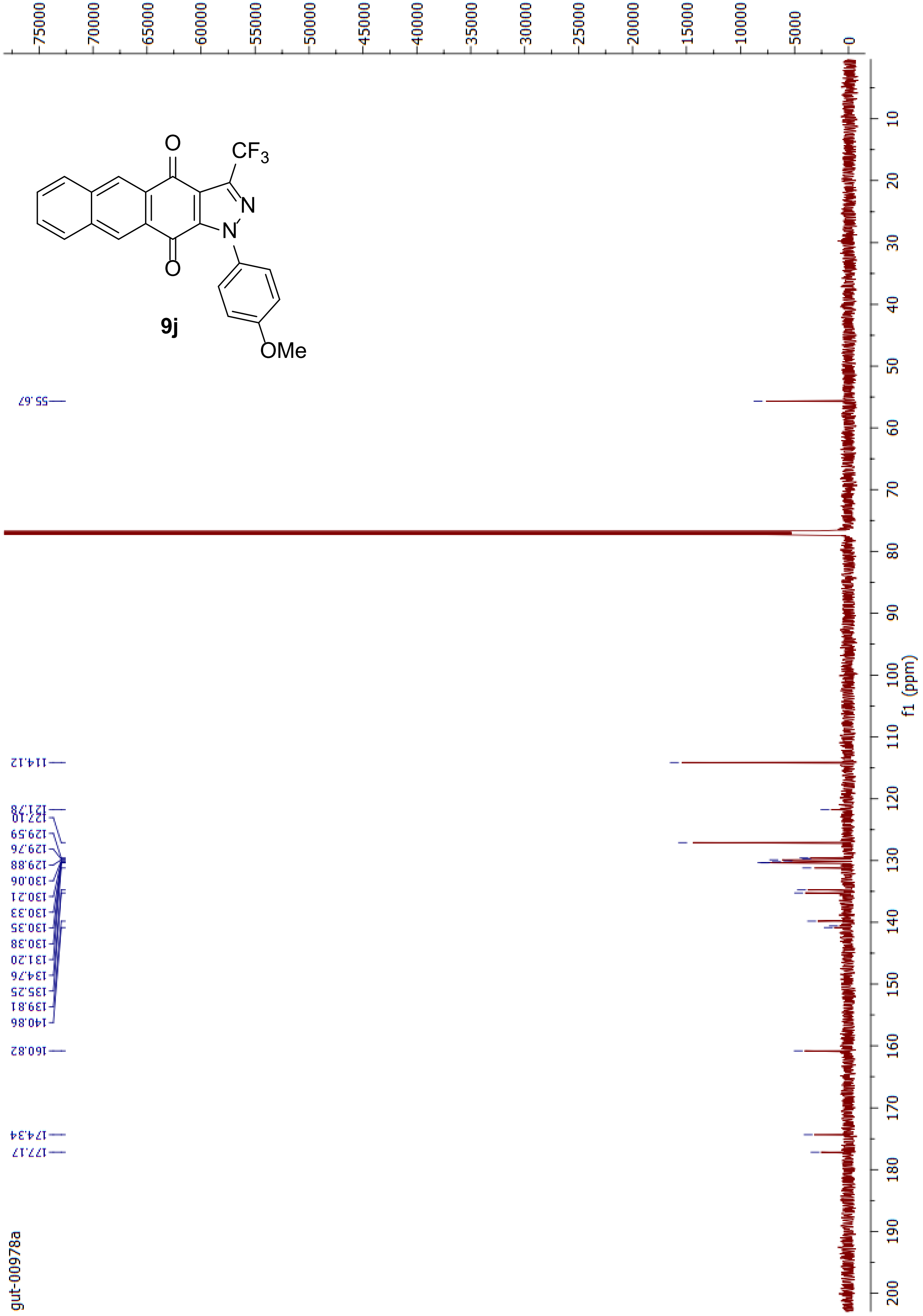

Figure S20. The ^{13}C NMR (151 MHz, $CDCl_3$) spectrum for compound **9j**.

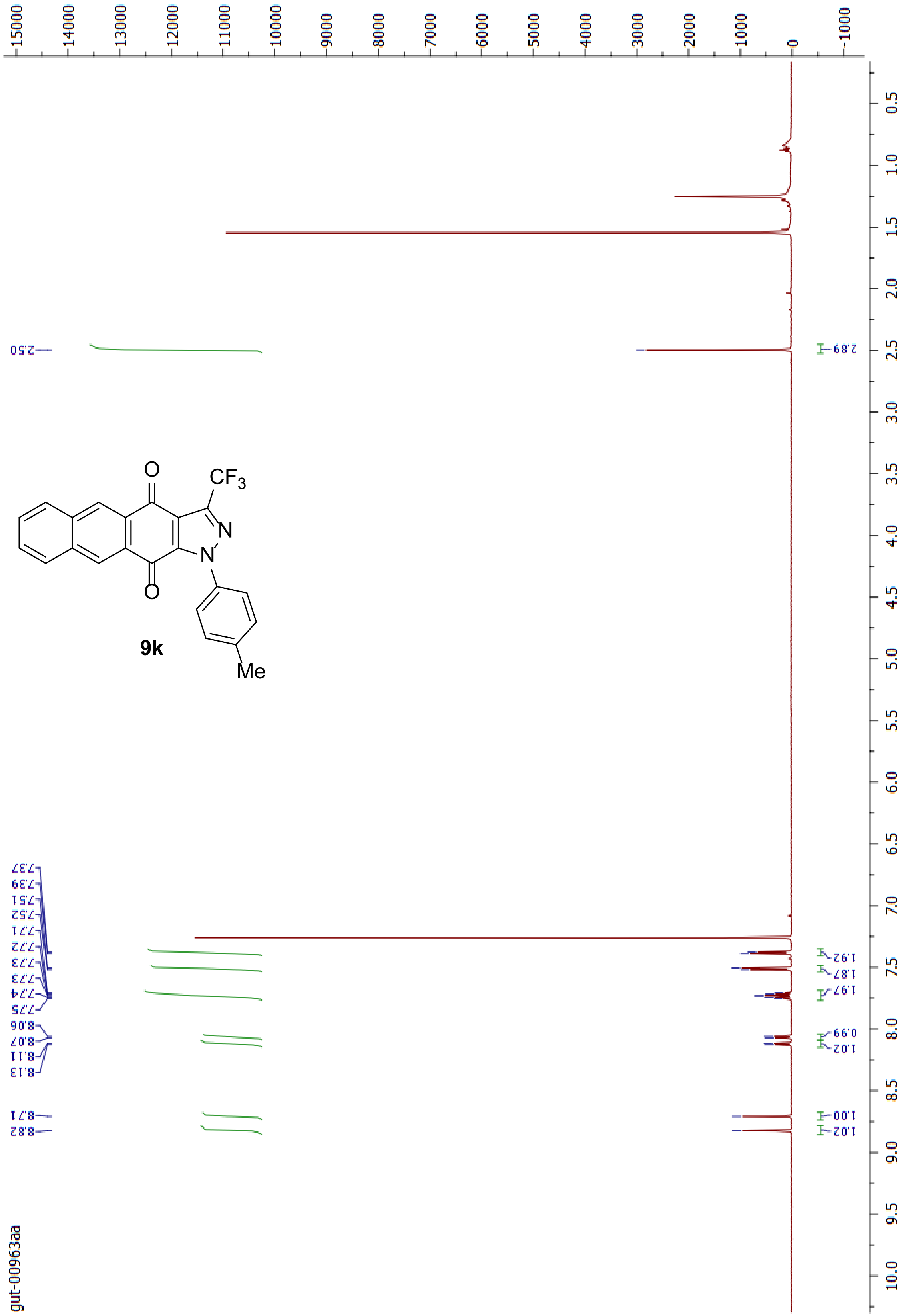

Figure S21. The ^{1}H NMR (600 MHz, $CDCl_3$) spectrum for compound **9k**.

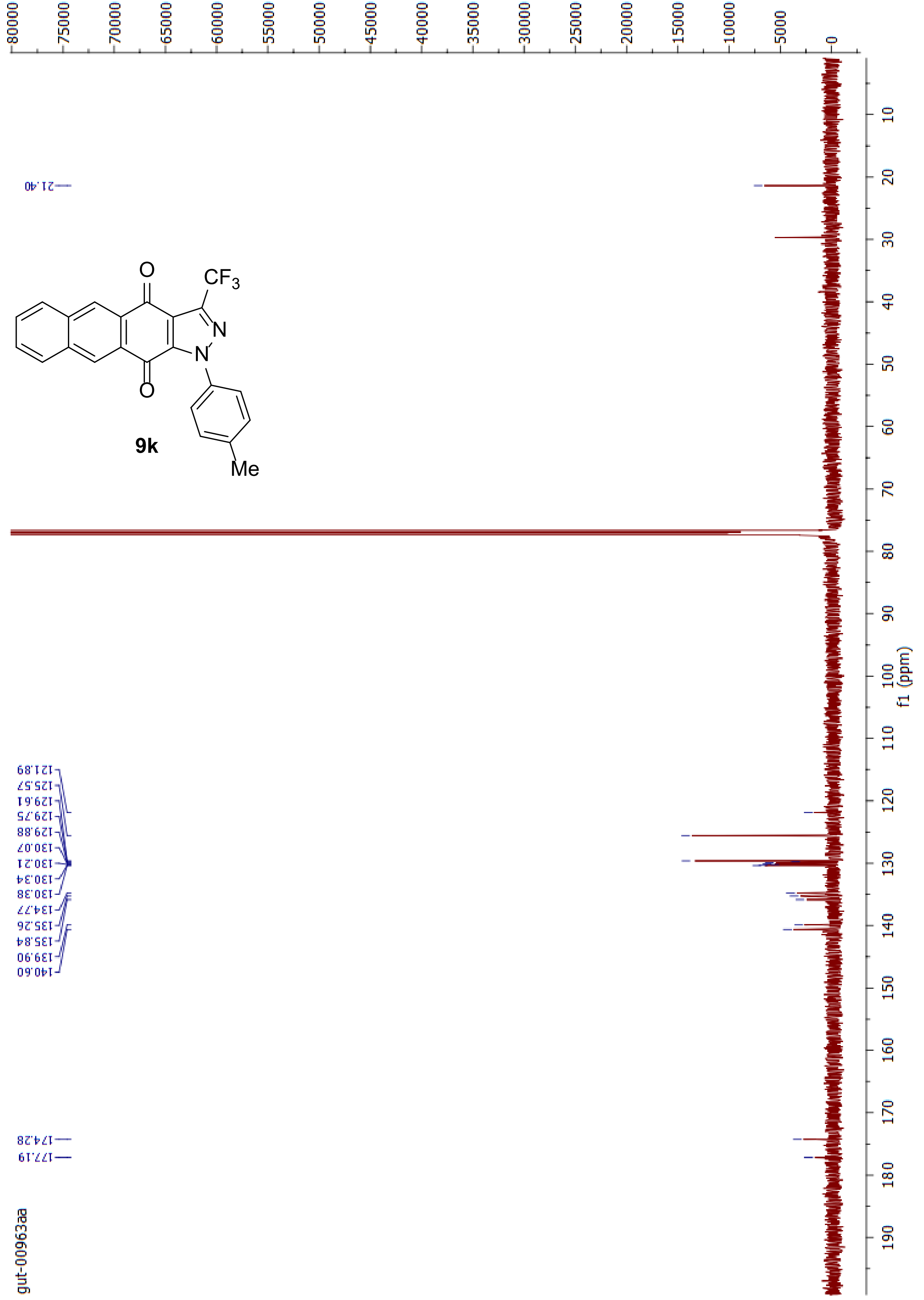

Figure S22. The ^{13}C NMR (151 MHz, $CDCl_3$) spectrum for compound **9k**.

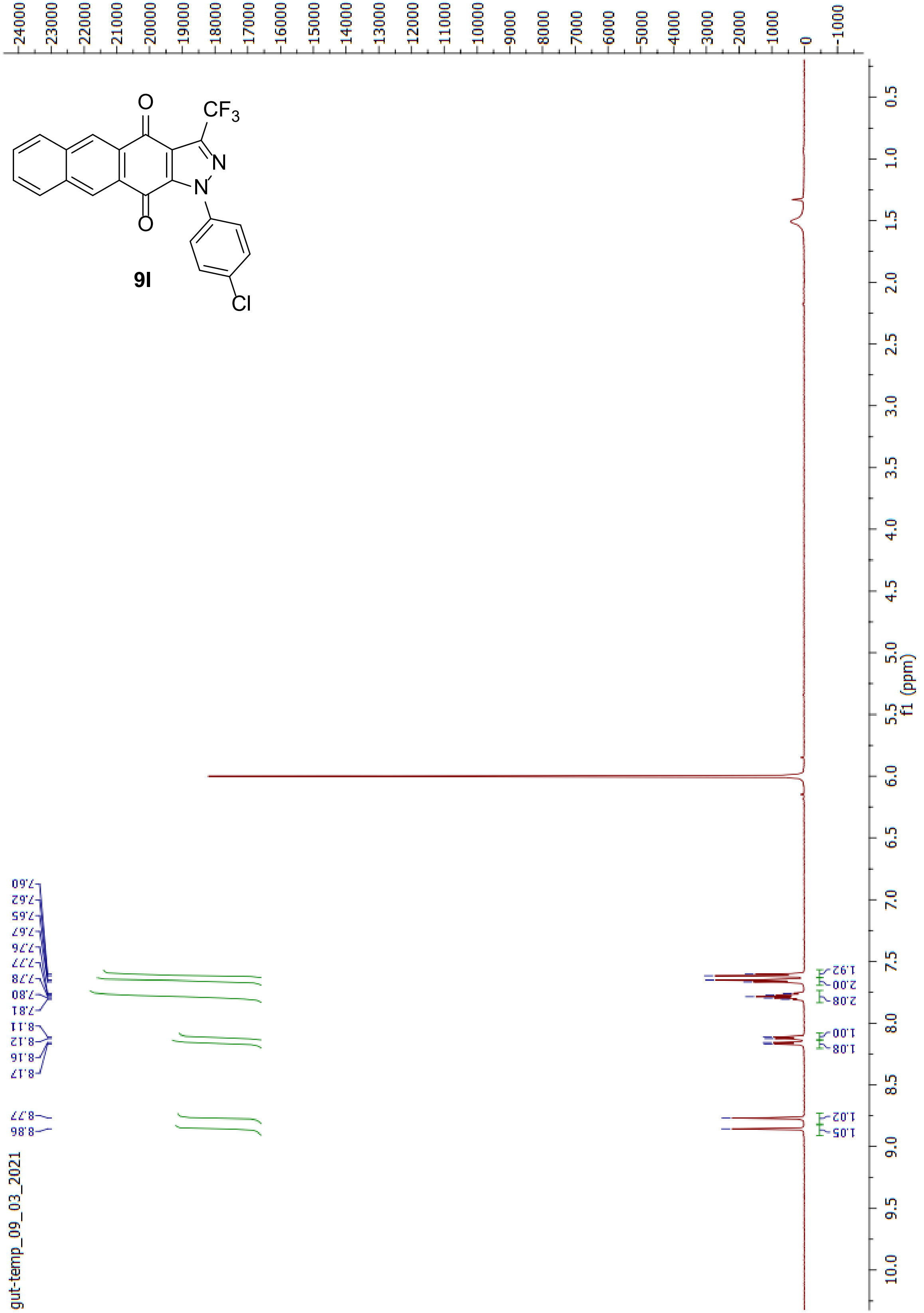

Figure S23. The ^1H NMR (600 MHz, $C_2D_2Cl_4$, 70°C) spectrum for compound **9l**.

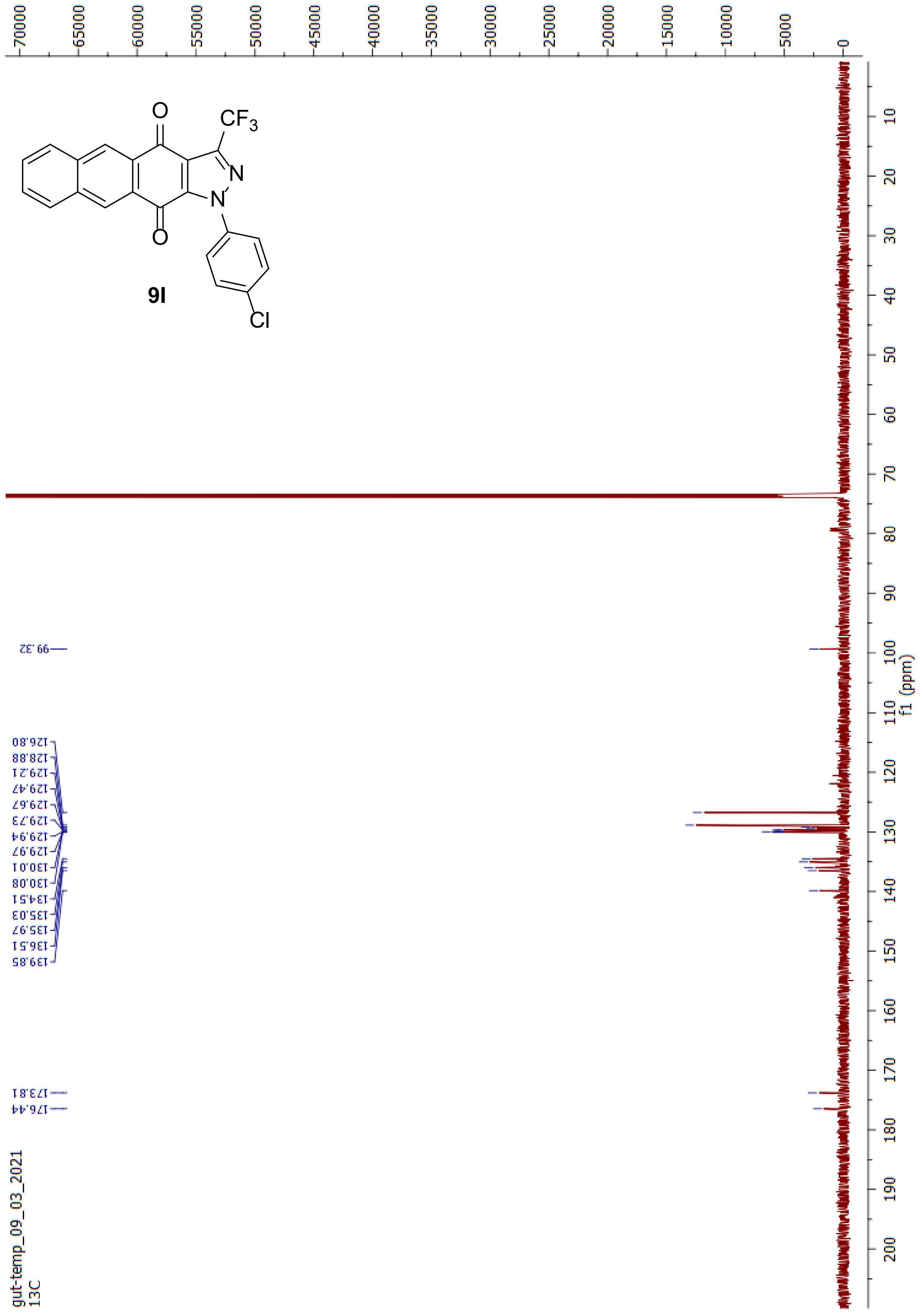

Figure S24. The ^{13}C NMR (151 MHz, $C_2D_2Cl_4$, 70°C) spectrum for compound **9l**.

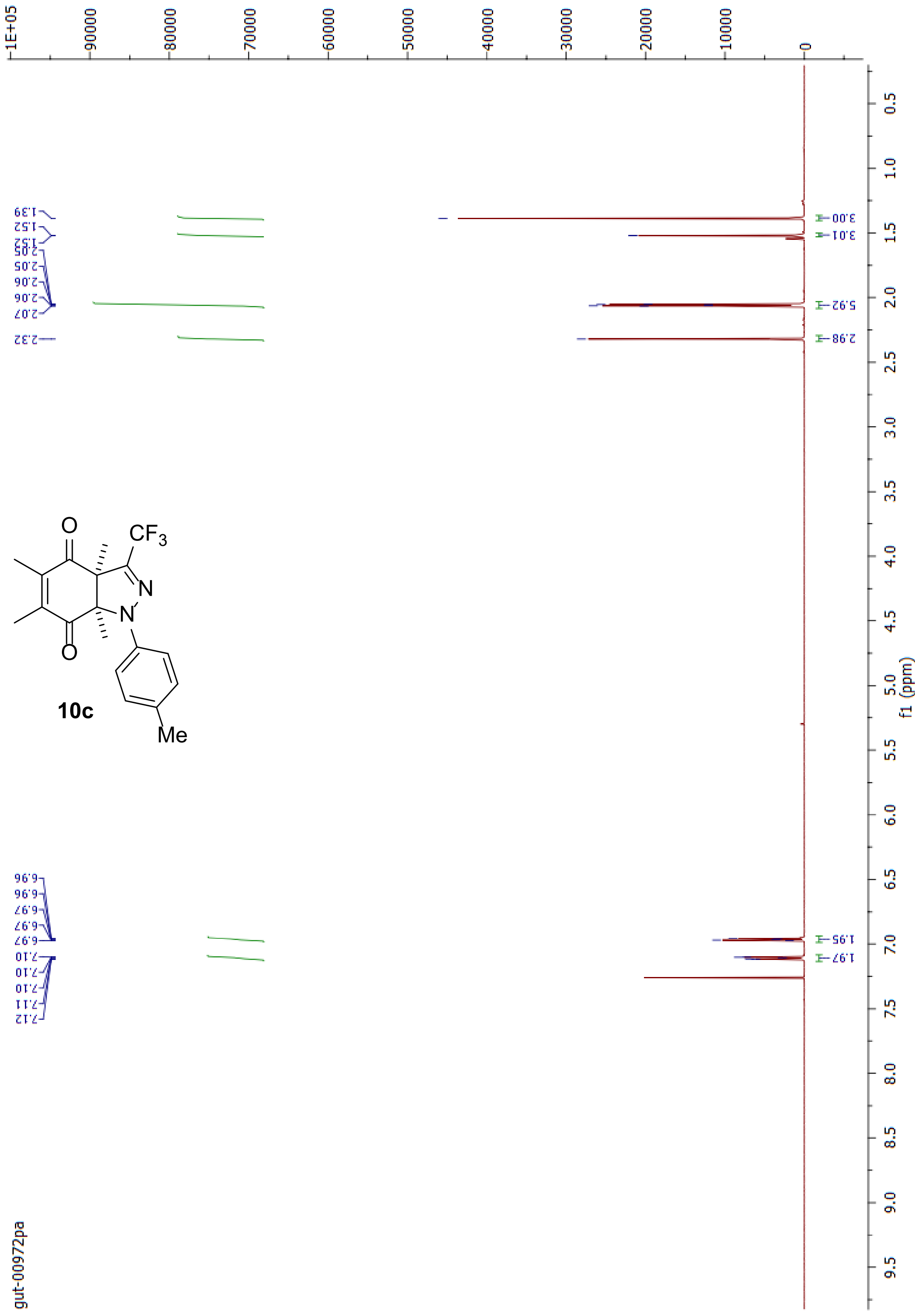

Figure S25. The ^{1}H NMR (600 MHz, $CDCl_3$) spectrum for compound **10c**.

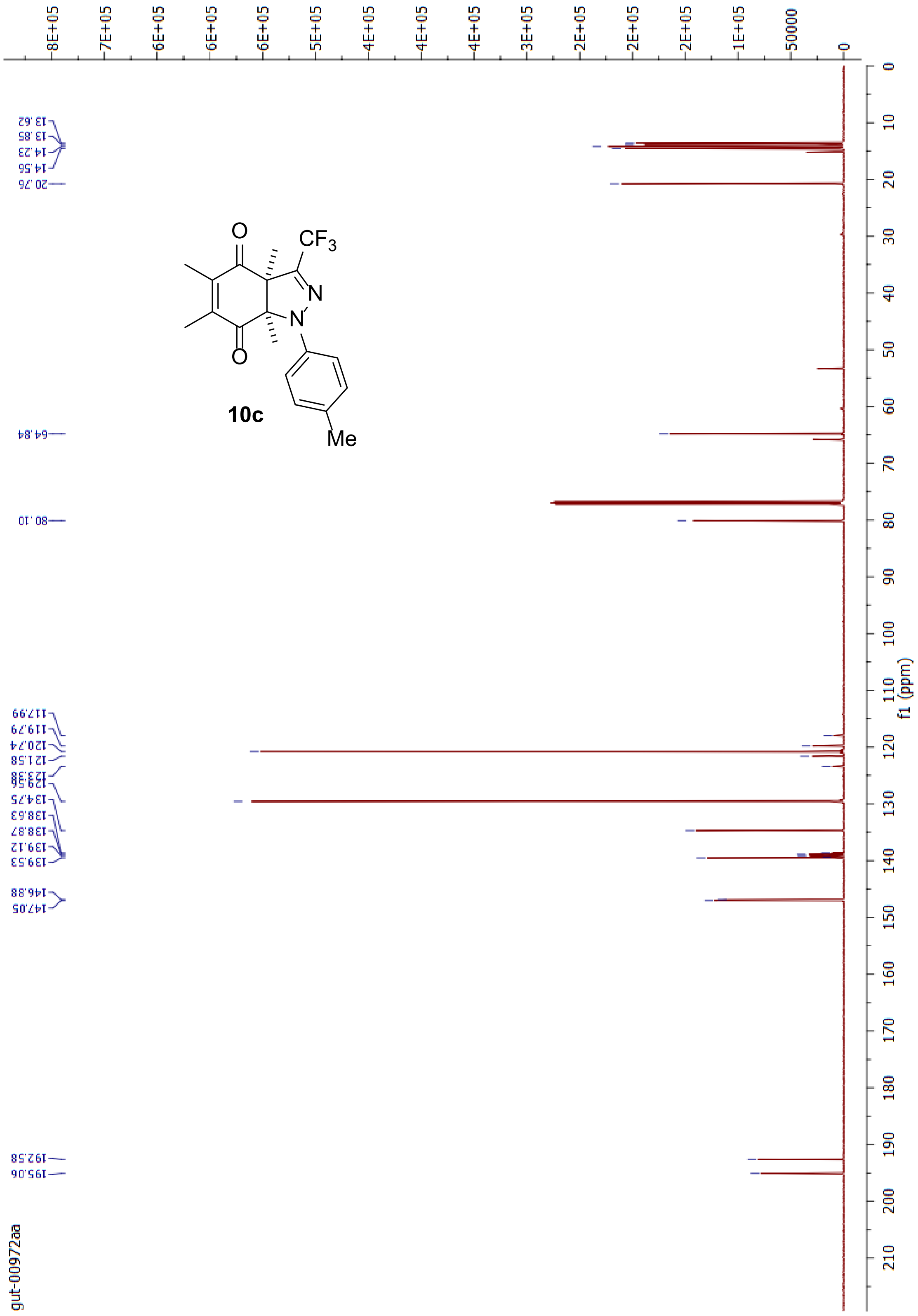

Figure S26. The ^{13}C NMR (151 MHz, $CDCl_3$) spectrum for compound **10c**.

UV–Vis measurements

UV–Vis spectra for **9a**–**9l** were recorded in spectroscopic-grade dichloromethane at concentrations in a range of 0.5–4.2 × 10^{-5} M and fitted to the Beer–Lambert law.

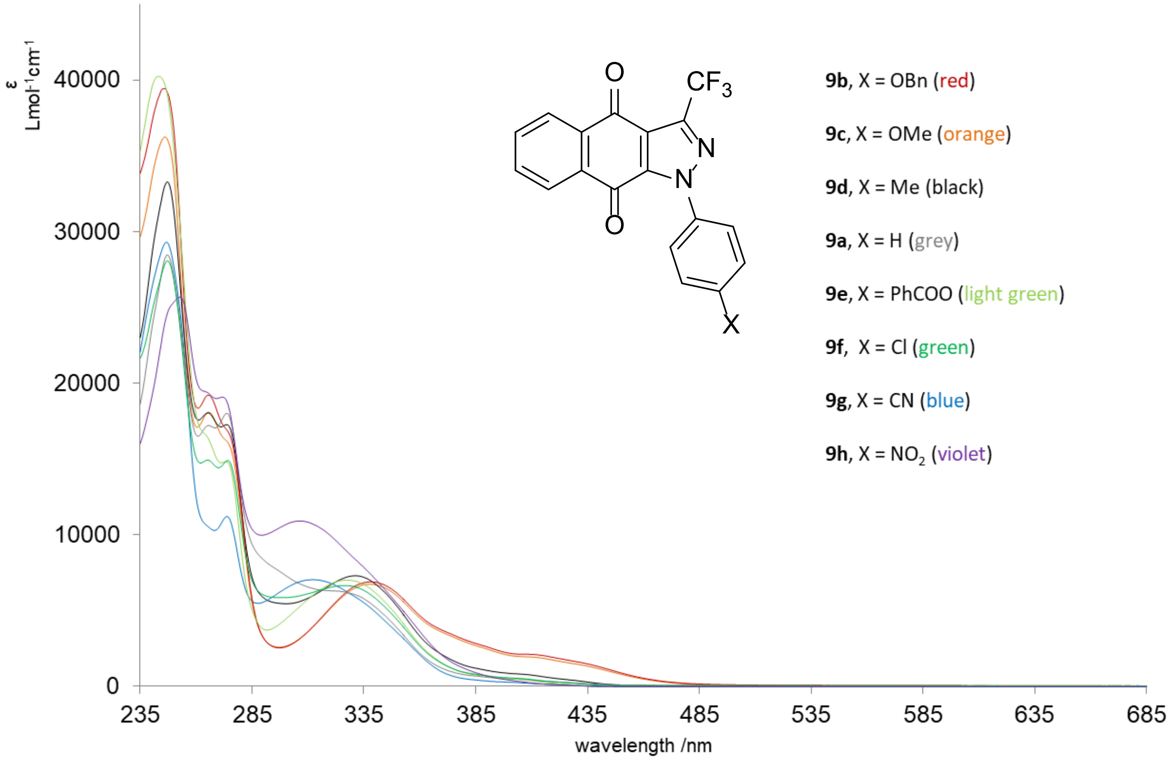

Figure S27. Collected data of electronic absorption spectra for naphtoquinone-fused pyrazoles **9a**–**9h** taken in CH_2Cl_2.

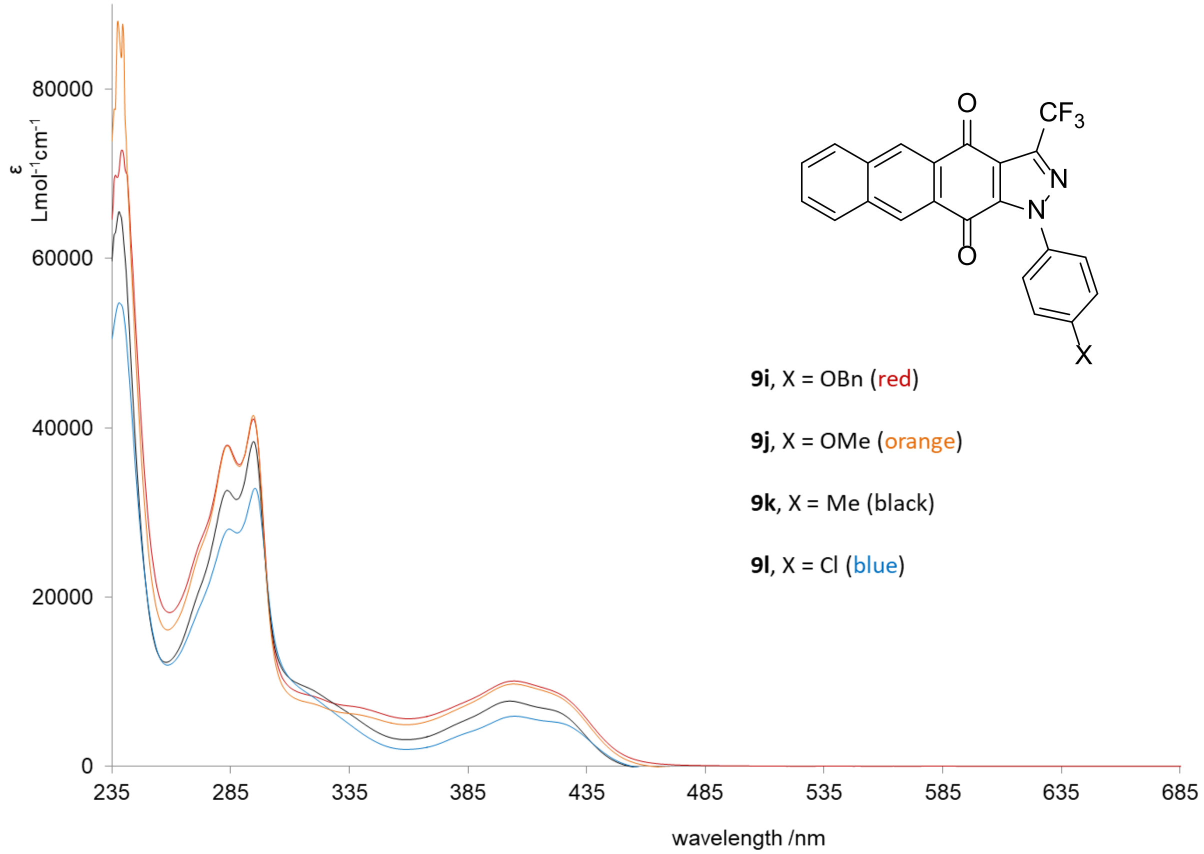

Figure S28. Electronic absorption spectra for pyrazoles **9i–9l** derived from 1,4-anthraquinone (in CH_2Cl_2).

X-ray data of pyrazole 9d

Table S1. Crystal data and structure refinement for exp_276_auto.

Identification code	exp_276_auto
Empirical formula	C19H11F3N2O2
Formula weight	356.30
Temperature/K	110.00(10)
Crystal system	orthorhombic
Space group	$Pna2_1$
a/Å	19.9357(2)
b/Å	4.243
c/Å	36.0046(3)
α/°	90
β/°	90
γ/°	90
Volume/Å^3	3045.81(4)
Z	8
ρ_{calc}mg/mm^3	1.554
μ/mm^{-1}	1.094
F(000)	1456.0
Crystal size/mm^3	0.507 × 0.138 × 0.101
2Θ range for data collection	8.872 to 136.996°
Index ranges	-24 ≤ h ≤ 23, -5 ≤ k ≤ 5, -43 ≤ l ≤ 43
Reflections collected	101855
Independent reflections	5588[R(int) = 0.0620]
Data/restraints/parameters	5588/1/471
Goodness-of-fit on F^2	1.041
Final R indexes [I>=2σ (I)]	R_1 = 0.0916, wR_2 = 0.2183
Final R indexes [all data]	R_1 = 0.0923, wR_2 = 0.2205
Largest diff. peak/hole / e Å^{-3}	1.07/-0.36
Flack parameter	0.41(4)

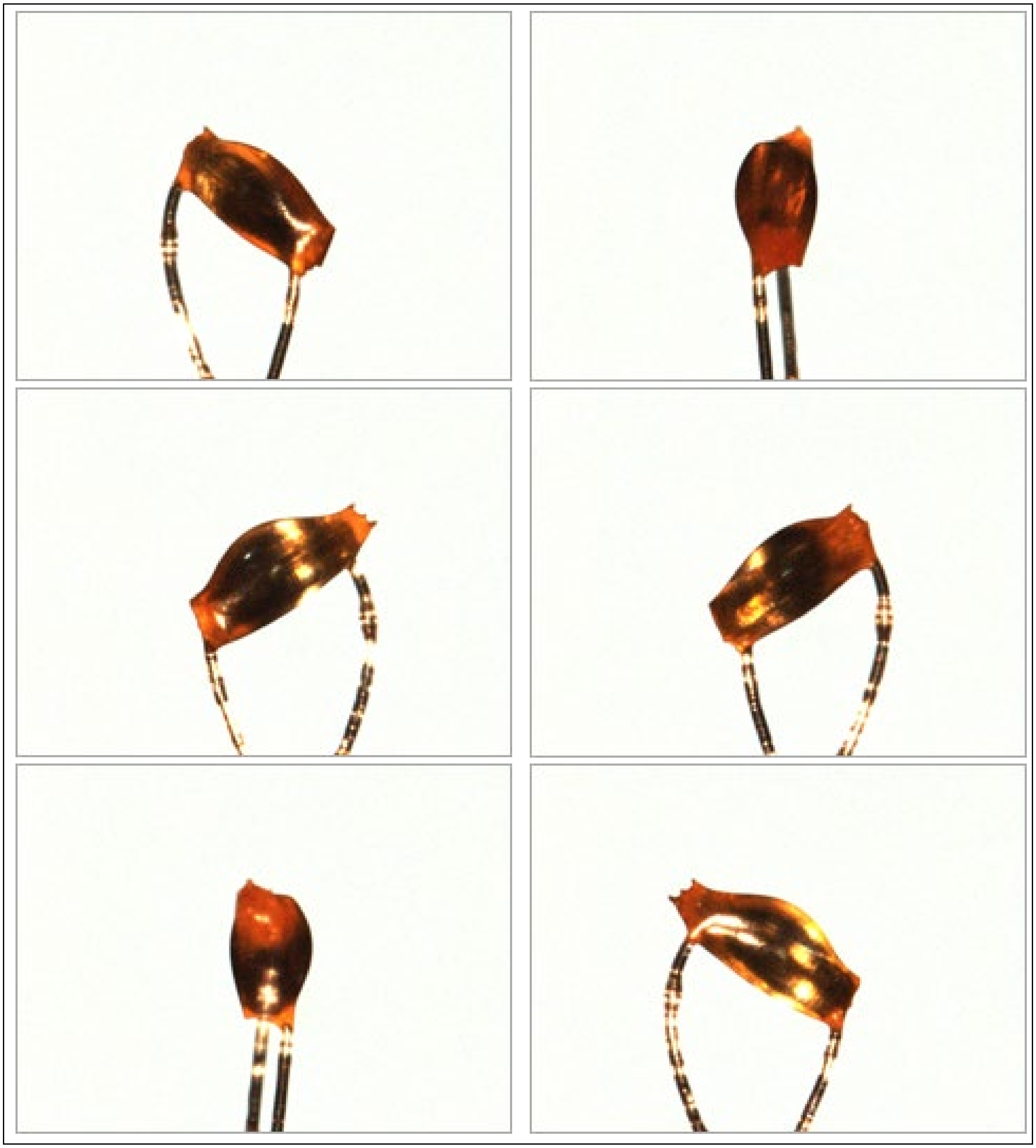

Figure S29. A set of sample photographs.

Table S2. Fractional atomic coordinates (× 10^4) and equivalent isotropic displacement parameters (Å^2 × 10^3) for exp_276_auto. U_{eq} is defined as 1/3 of the trace of the orthogonalized U_{IJ} tensor.

atom	*x*	*y*	*z*	*U*(eq)
F2	6103.0(16)	12031(8)	7536.0(9)	30.8(8)
F8	6666.7(17)	11707(9)	7031.0(10)	40.4(8)
F13	6730.5(18)	7952(9)	7437.7(13)	48.2(11)
F14	5753.4(19)	1967(9)	4062.3(15)	55.0(13)
O2	8483.7(19)	7594(9)	4821.7(10)	24.6(8)
F23	6376.0(17)	-2132(8)	3969.8(10)	33.5(8)
O3	5190(2)	7605(10)	7885.9(11)	28.9(9)
O1	7316(2)	2165(10)	3619.3(12)	32.3(9)
F35	5798.3(17)	-1757(10)	4469.4(10)	42.9(9)
O4	3982(2)	2354(10)	6690.5(12)	30.8(9)
N1	5767(2)	7891(10)	6720.4(13)	23.4(9)
C43	5146(2)	7036(13)	7238.7(15)	21.2(10)
N4	7248.1(19)	3684(9)	4878.5(12)	20.8(8)
N3	6696(2)	2047(10)	4787.2(12)	21.7(9)
N2	5208(2)	6304(10)	6631.6(12)	23.5(9)
C41	5739(2)	8363(12)	7084.0(14)	22.4(10)
C27	9110(3)	7301(14)	3480.7(16)	32.1(13)
C30	7583(3)	3242(13)	3893.9(14)	25.0(11)
C55	4927(2)	3877(12)	5505.0(14)	23.8(10)
C46	3927(2)	3452(13)	7340.2(13)	22.9(10)
C38	7610(3)	7017(15)	6407.9(16)	31.2(12)
C32	7353(2)	4545(12)	5258.3(13)	20.5(10)
C28	8240(2)	6228(12)	4551.3(13)	22.7(10)
C29	7640(2)	4280(12)	4576.1(13)	21.8(10)
C56	5484(3)	2736(12)	5702.3(15)	24.9(11)
C45	4248(2)	4814(12)	7650.6(14)	23.9(10)
C42	4825(2)	5689(11)	6934.2(14)	22.1(9)
C39	6163(2)	-79(11)	4224.6(15)	26.9(11)
C53	4548(2)	6636(13)	6059.2(13)	23.1(10)

C31	6733(2)	1579(13)	4417.3(15)	25.4(10)
C47	4210(2)	3732(11)	6954.1(14)	22.0(9)
C25	8532(2)	6475(12)	4172.9(14)	23.8(10)
C23	8500(3)	5521(13)	3509.8(14)	28.2(11)
C36	7907(2)	3412(12)	5448.5(17)	27.9(11)
C24	8220(2)	5162(11)	3859.7(14)	23.0(10)
C54	4466(2)	5794(12)	5684.7(14)	26.5(10)
C48	3333(3)	1856(15)	7384.1(14)	28.4(11)
C52	5102(2)	5458(12)	6246.5(14)	23.2(10)
C34	6977(3)	7185(14)	5801.2(16)	31.1(13)
C50	3387(3)	2749(13)	8053.8(15)	27.3(12)
C57	5573(3)	3555(14)	6075.5(13)	27.2(11)
C44	4887(3)	6614(13)	7621.5(14)	25.4(11)
C37	7987(2)	4195(13)	5821.3(13)	24.8(10)
C33	6884(3)	6409(13)	5432.4(15)	28.9(11)
C40	6304(2)	9992(11)	7268.8(14)	24.3(10)
C26	9123(3)	8235(14)	4127.6(16)	29.6(12)
C35	7528(2)	6108(12)	6001.9(14)	24.5(10)
C4	9403(3)	8624(15)	3776.9(16)	31.9(12)
C49	3053(3)	1503(15)	7738.4(16)	32.1(12)
C51	3959(3)	4416(13)	8001.1(15)	30.4(11)
C20	7318(3)	2895(12)	4272.9(15)	22.5(11)
C22	4838(3)	2937(14)	5103.1(16)	30.0(13)

Table S3. Anisotropic displacement parameters (Å^2 × 10^3) for exp_276_auto. The anisotropic displacement factor exponent takes the form: $-2\pi^2[h^2a^{*2}U_{11}+2hka^*b^*U_{12}+\ldots]$.

atom	*U*11	*U*22	*U*33	*U*23	*U*13	*U*12
F2	32.0(17)	37.3(18)	23.0(15)	-6.1(14)	-1.4(13)	-3.0(13)
F8	37.1(18)	45(2)	38.9(18)	-7.8(16)	6.4(15)	-17.2(15)
F13	35.9(18)	32.1(18)	76(3)	-1.2(19)	-34(2)	-0.7(14)
F14	41(2)	28.7(19)	96(4)	-2(2)	-40(2)	6.0(15)
O2	27.3(17)	37(2)	9.7(15)	-5.8(14)	-4.2(14)	-6.1(14)

F23	36.8(17)	34.0(17)	29.6(16)	-5.9(14)	-2.3(14)	-9.9(13)
O3	35(2)	36(2)	16.1(18)	-0.4(14)	-10.1(16)	-8.1(16)
O1	35(2)	41(2)	21.4(19)	-4.6(15)	0.2(16)	-8.0(16)
F35	34.8(17)	58(2)	36.0(18)	-8.4(17)	6.4(15)	-22.1(16)
O4	26.5(19)	40(2)	26(2)	-3.7(16)	2.6(16)	-7.7(15)
N1	18(2)	22(2)	30(2)	0.3(17)	0.5(17)	-1.1(15)
C43	15(2)	22(2)	27(3)	1(2)	0.7(18)	2.5(18)
N4	19.3(18)	23.0(19)	20.2(19)	-2.0(17)	-0.5(14)	-2.5(15)
N3	20(2)	26(2)	18.2(19)	-0.6(17)	-4.0(16)	-0.7(15)
N2	22.5(19)	26(2)	22.3(19)	1.9(18)	-0.3(16)	1.4(17)
C41	23(2)	19(2)	25(2)	-1(2)	-2.1(19)	-2.2(18)
C27	37(3)	38(3)	21(3)	1(2)	6(2)	2(2)
C30	27(3)	29(3)	18(2)	-4(2)	-0.5(19)	2(2)
C55	26(2)	20(2)	26(2)	0.1(19)	1.9(19)	1.0(19)
C46	21(2)	30(3)	17(2)	0(2)	-0.9(18)	-1.6(19)
C38	23(3)	46(3)	24(3)	-4(2)	0(2)	5(2)
C32	20(2)	22(2)	19(2)	1.6(17)	-1.0(17)	-4.1(18)
C28	23(2)	26(2)	19(2)	1(2)	-1.5(18)	3.9(19)
C29	22(2)	25(2)	18(2)	-1.7(18)	1.0(17)	2.1(19)
C56	24(3)	29(2)	22(3)	-1.4(19)	4(2)	8(2)
C45	28(2)	24(2)	20(2)	-1.3(18)	-2.2(19)	1.0(19)
C42	20(2)	21(2)	25(2)	3(2)	-3.1(17)	1.2(18)
C39	24(2)	22(2)	35(3)	-6(2)	-9.4(19)	-2(2)
C53	18(2)	37(3)	15(2)	-1(2)	-1.3(16)	1(2)
C31	20(2)	26(2)	31(3)	3(2)	-7.5(19)	3.8(19)
C47	24(2)	18(2)	25(2)	-1.3(19)	-3.4(19)	0.8(19)
C25	25(2)	18(2)	27(2)	-3(2)	-1(2)	5.0(19)
C23	33(3)	30(3)	21(2)	-2(2)	-2(2)	-1(2)
C36	19(2)	26(2)	38(3)	-2(3)	2(2)	7(2)
C24	23(2)	21(2)	25(2)	-2.2(19)	-1.6(19)	2.2(19)
C54	21(2)	29(3)	30(3)	-1(2)	-2.3(19)	2(2)
C48	27(3)	40(3)	19(2)	1(2)	2.6(19)	-2(2)
C52	22(2)	26(2)	22(2)	-2.9(19)	0.3(17)	-0.8(19)

C34	32(3)	35(3)	26(3)	0(2)	-2(2)	12(2)
C50	35(3)	32(3)	15(2)	-1.4(19)	7(2)	-3(2)
C57	27(2)	36(3)	19(2)	0(2)	-0.6(19)	6(2)
C44	25(2)	26(2)	25(3)	1(2)	-3.2(19)	2(2)
C37	18(2)	30(3)	26(2)	1.0(19)	-5.1(17)	0(2)
C33	25(2)	27(3)	35(3)	2(2)	-1(2)	7(2)
C40	25(2)	17(2)	30(3)	4.5(18)	-1.0(18)	2.6(19)
C26	24(2)	34(3)	31(3)	-10(2)	-3(2)	-3(2)
C35	26(2)	22(2)	26(2)	3(2)	0.9(19)	-2.9(19)
C4	27(3)	32(3)	36(3)	-2(3)	2(2)	-4(2)
C49	26(2)	43(3)	28(3)	1(2)	3(2)	-3(2)
C51	36(3)	33(3)	23(2)	-3(2)	0(2)	0(2)
C20	23(3)	29(3)	15(2)	-2.4(19)	-5.6(18)	-1.0(19)
C22	34(3)	36(3)	20(3)	-3(2)	-1(2)	2(2)

Table S4. Bond lengths for exp_276_auto.

atom	atom	length/Å		atom	atom	length/Å
F2	C40	1.355(6)		C46	C45	1.411(7)
F8	C40	1.336(6)		C46	C47	1.505(7)
F13	C40	1.357(6)		C46	C48	1.374(8)
F14	C39	1.327(6)		C38	C35	1.521(8)
O2	C28	1.233(7)		C32	C36	1.386(7)
F23	C39	1.335(6)		C32	C33	1.375(7)
O3	C44	1.203(7)		C28	C29	1.458(7)
O1	C30	1.212(7)		C28	C25	1.485(7)
F35	C39	1.346(6)		C29	C20	1.396(7)
O4	C47	1.204(7)		C56	C57	1.400(7)
N1	N2	1.342(6)		C45	C44	1.489(7)
N1	C41	1.326(7)		C45	C51	1.397(7)
C43	C41	1.422(7)		C42	C47	1.484(7)
C43	C42	1.392(8)		C39	C31	1.506(7)
C43	C44	1.483(8)		C53	C54	1.404(7)

N4	N3	1.342(6)		C53	C52	1.387(7)
N4	C32	1.431(6)		C31	C20	1.393(7)
N4	C29	1.363(6)		C25	C24	1.404(7)
N3	C31	1.348(7)		C25	C26	1.403(8)
N2	C42	1.355(6)		C23	C24	1.386(8)
N2	C52	1.448(6)		C36	C37	1.392(8)
C41	C40	1.480(7)		C48	C49	1.400(7)
C27	C23	1.436(8)		C52	C57	1.383(7)
C27	C4	1.339(9)		C34	C33	1.381(8)
C30	C24	1.514(7)		C34	C35	1.391(7)
C30	C20	1.471(7)		C50	C49	1.418(8)
C55	C56	1.405(8)		C50	C51	1.355(8)
C55	C54	1.387(7)		C37	C35	1.386(7)
C55	C22	1.511(7)		C26	C4	1.391(8)

Table S5. Bond angles for exp_276_auto.

atom	atom	atom	angle/˚		atom	atom	atom	angle/˚
C41	N1	N2	106.0(4)		F35	C39	C31	110.7(4)
C41	C43	C44	134.6(5)		C52	C53	C54	117.9(5)
C42	C43	C41	103.7(5)		N3	C31	C39	118.9(5)
C42	C43	C44	121.5(5)		N3	C31	C20	110.8(4)
N3	N4	C32	119.1(4)		C20	C31	C39	130.3(5)
N3	N4	C29	111.7(4)		O4	C47	C46	123.3(5)
C29	N4	C32	129.2(4)		O4	C47	C42	123.1(5)
N4	N3	C31	105.9(4)		C42	C47	C46	113.5(4)
N1	N2	C42	111.9(4)		C24	C25	C28	122.4(4)
N1	N2	C52	118.3(4)		C26	C25	C28	118.2(5)
C42	N2	C52	129.8(4)		C26	C25	C24	119.3(5)
N1	C41	C43	111.2(5)		C24	C23	C27	117.7(5)
N1	C41	C40	118.8(5)		C32	C36	C37	119.0(5)
C43	C41	C40	129.9(4)		C25	C24	C30	121.3(5)
C4	C27	C23	122.1(5)		C23	C24	C30	118.1(5)
O1	C30	C24	120.3(5)		C23	C24	C25	120.6(5)

O1	C30	C20	124.1(5)		C55	C54	C53	121.4(5)
C20	C30	C24	115.6(4)		C46	C48	C49	120.1(5)
C56	C55	C22	119.1(5)		C53	C52	N2	119.5(4)
C54	C55	C56	119.3(5)		C57	C52	N2	118.2(4)
C54	C55	C22	121.6(5)		C57	C52	C53	122.3(5)
C45	C46	C47	122.0(4)		C33	C34	C35	121.8(5)
C48	C46	C45	120.1(5)		C51	C50	C49	118.5(5)
C48	C46	C47	117.9(5)		C52	C57	C56	119.1(5)
C36	C32	N4	120.0(4)		O3	C44	C43	121.2(5)
C33	C32	N4	118.9(4)		O3	C44	C45	123.6(5)
C33	C32	C36	121.1(5)		C43	C44	C45	115.2(4)
O2	C28	C29	122.8(4)		C35	C37	C36	121.1(4)
O2	C28	C25	122.5(5)		C32	C33	C34	119.0(5)
C29	C28	C25	114.7(4)		F2	C40	F13	105.9(4)
N4	C29	C28	128.6(4)		F2	C40	C41	113.1(4)
N4	C29	C20	106.4(4)		F8	C40	F2	105.5(4)
C20	C29	C28	124.6(4)		F8	C40	F13	107.2(4)
C57	C56	C55	120.0(5)		F8	C40	C41	112.2(4)
C46	C45	C44	122.8(5)		F13	C40	C41	112.4(4)
C51	C45	C46	118.7(5)		C4	C26	C25	120.4(5)
C51	C45	C44	118.5(5)		C34	C35	C38	120.0(5)
C43	C42	C47	124.9(5)		C37	C35	C38	121.9(5)
N2	C42	C43	107.2(4)		C37	C35	C34	118.1(5)
N2	C42	C47	127.7(5)		C27	C4	C26	119.9(5)
F14	C39	F23	108.7(5)		C48	C49	C50	120.2(5)
F14	C39	F35	107.6(4)		C50	C51	C45	122.4(5)
F14	C39	C31	111.2(4)		C29	C20	C30	121.2(4)
F23	C39	F35	106.0(4)		C31	C20	C30	133.4(5)
F23	C39	C31	112.4(4)		C31	C20	C29	105.2(5)

Table S6. Hydrogen atom coordinates (Å × 10^4) and isotropic displacement parameters (Å^2 × 10^3) for exp_276_auto.

atom	*x*	*y*	*z*	*U*(eq)
H27	9312.51	7548.75	3243.66	38
H38A	7602.71	9317.88	6431.21	47

H38B	8038.06	6205.17	6501.03	47
H38C	7241.1	6114	6553.24	47
H56	5800.87	1408.92	5582.21	30
H53	4234.29	7970.01	6180.31	28
H23	8294.75	4620.29	3296.64	34
H36	8227.18	2120	5326.18	33
H54	4087.81	6552.67	5551.81	32
H48	3111.31	991.09	7173.94	34
H34	6656.5	8491.06	5921.3	37
H50	3213.59	2421.89	8296.51	33
H57	5952.4	2815.13	6209.65	33
H37	8362.89	3404.65	5954.15	30
H33	6502.31	7151.49	5300.95	35
H26	9331.77	9166.6	4337.71	35
H4	9801.52	9825.26	3747.46	38
H49	2638.83	426.78	7767.59	39
H51	4171.72	5348.08	8210.13	36
H22A	5277.52	2827.48	4982.06	45
H22B	4558.64	4505.03	4976.41	45
H22C	4619.68	870.72	5090.23	45

Experimental

After crystallization, the obtained crystals were relatively large and well-shaped (light-range blocks). However, they appeared to be extremely fragile and after being touched, they were rapidly falling apart into smaller parts (needles) of high mosaicity. Due to that reason, our measurement merely fulfils requirements of expected X-ray experiment quality. Consequently, the best possible crystal sample of $C_{19}H_{11}F_3N_2O_2$ suitable for an X-ray experiment was selected and measured on a XtaLAB Synergy, Dualflex, HyPix diffractometer. The crystal was kept at 110.00(10) K during data collection. Using Olex2

[1], the structure was solved with the Superflip [2] structure solution program using charge flipping and refined with the SHELXL [3] refinement package using least squares minimization.

Crystal structure determination

Crystal Data for $C_{19}H_{11}F_3N_2O_2$ (M = 356.30 g/mol): orthorhombic, space group $Pna2_1$ (no. 33), a = 19.9357(2) Å, b = 4.243 Å, c = 36.0046(3) Å, V = 3045.81(4) Å^3, Z = 8, T = 110.00(10) K, μ(Cu Kα) = 1.094 mm^{-1}, $Dcalc$ = 1.554 g/cm^3, 101855 reflections measured (8.872° ≤ 2Θ ≤ 136.996°), 5588 unique (R_{int} = 0.0620, R_{sigma} = 0.0194), which were used in all calculations. The final R_1 was 0.0916 (I > 2σ(I)) and wR_2 was 0.2205 (all data).

Refinement model description

Number of restraints - 1, number of constraints - unknown.

Details:

1. Fixed Uiso

At 1.2 times of:

All C(H) groups

At 1.5 times of:

All C(H,H,H) groups

2.a Aromatic/amide H refined with riding coordinates:

C27(H27), C56(H56), C53(H53), C23(H23), C36(H36), C54(H54), C48(H48), C34(H34), C50(H50), C57(H57), C37(H37), C33(H33), C26(H26), C4(H4), C49(H49),

C51(H51)

2.b Idealised Me refined as rotating group:

C38(H38A,H38B,H38C), C22(H22A,H22B,H22C)

References

[1] Dolomanov, O.V., Bourhis, L.J., Gildea, R.J, Howard, J.A.K. & Puschmann, H. (2009), J. Appl. Cryst. 42, 339-341.

[2] Palatinus, L. & Chapuis, G. (2007). J. Appl. Cryst., 40, 786-790; Palatinus, L. & van der Lee, A. (2008). J. Appl. Cryst. 41, 975-984; Palatinus, L., Prathapa, S. J. & van Smaalen, S. (2012). J. Appl. Cryst. 45, 575-580.

[3] Sheldrick, G.M. (2015). Acta Cryst. C71, 3-8.
